# Prognostic Implications of Immune-Related Gene Pairs Signatures in Bladder Cancer

**DOI:** 10.1155/2021/5345181

**Published:** 2021-07-26

**Authors:** Hui Xiong, Hui Gao, Jinding Hu, Yun Dai, Hanbo Wang, Min Fu, Taiguo Qi, Lianjun Li, Qinghua Xia, Xunbo Jin, Zilian Cui, Weiting Kang

**Affiliations:** ^1^Department of Urology, Shandong Provincial Hospital Affiliated to Shandong First Medical University, Jinan, Shandong 250021, China; ^2^Department of Urology, Liaocheng People's Hospital, Liaocheng, Shandong 252000, China; ^3^Department of Urology, The Second People's Hospital of Liaocheng, Liaocheng, Shandong 252600, China; ^4^Department of Urology, The Second Hospital of Liaocheng Affiliated to Shandong First Medical University, Liaocheng, Shandong 252600, China; ^5^Department of Ultrasound, Shandong Provincial Hospital Affiliated to Shandong First Medical University, Jinan, Shandong 250021, China; ^6^Department of Ultrasound, Shandong Provincial Hospital, Cheeloo College of Medicine, Shandong University, Jinan, Shandong 250021, China; ^7^Department of Urology, Shandong Provincial Hospital, Cheeloo College of Medicine, Shandong University, Jinan, Shandong 250021, China

## Abstract

Compelling evidence indicates that immune function is correlated with the prognosis of bladder cancer (BC). Here, we aimed to develop a clinically translatable immune-related gene pairs (IRGPs) prognostic signature to estimate the overall survival (OS) of bladder cancer. From the 251 prognostic-related IRGPs, 37 prognostic-related IRGPs were identified using LASSO regression. We identified IRGPs with the potential to be prognostic markers. The established risk scores divided BC patients into high and low risk score groups, and the survival analysis showed that risk score was related to OS in the TCGA-training set (*p* < 0.001; HR = 7.5 [5.3, 10]). ROC curve analysis showed that the AUC for the 1-year, 3-year, and 5-year follow-up was 0.820, 0.883, and 0.879, respectively. The model was verified in the TCGA-testing set and external dataset GSE13507. Multivariate analysis showed that risk score was an independent prognostic predictor in patients with BC. In addition, significant differences were found in gene mutations, copy number variations, and gene expression levels in patients with BC between the high and low risk score groups. Gene set enrichment analysis showed that, in the high-risk score group, multiple immune-related pathways were inhibited, and multiple mesenchymal phenotype-related pathways were activated. Immune infiltration analysis revealed that immune cells associated with poor prognosis of BC were upregulated in the high-risk score group, whereas immune cells associated with a better prognosis of BC were downregulated in the high-risk score group. Other immunoregulatory genes were also differentially expressed between high and low risk score groups. A 37 IRGPs-based risk score signature is presented in this study. This signature can efficiently classify BC patients into high and low risk score groups. This signature can be exploited to select high-risk BC patients for more targeted treatment.

## 1. Introduction

Bladder cancer is the most common malignant tumor of the urinary system with high morbidity and mortality [1]. It is estimated that, in 2020, there would be approximately 81,400 new cases of bladder cancer cases and more than 17,980 deaths due to kidney cancer in the United States [2]. At the time of initial diagnosis, approximately 75% of bladder cancer patients are non-muscle invasive bladder cancer (NMIBC), and about 25% of the patients are muscle infiltrating bladder cancer (MIBC), or metastatic disease [3, 4]. NMIBC is commonly treated locally through intravesical chemotherapy or immunotherapy in combination with TURBT. However, most NMIBC relapses within 6–12 months, and 10%–15% of patients may progress to invasive or metastatic disease [5]. Overall, the 5-year survival rate of bladder cancer at all stages does not exceed 20% [6].

The tumor microenvironment (TME) is primarily composed of tumor cells, stroma, and invading immune cells. The immune cells in TME play a critical role in suppressing cancer or promoting tumors. Notably, the CD8 + T cells and natural killer cells play an antitumor function, whereas tumor-associated macrophages and regulatory T cells serve as tumor promoters [7, 8]. Recently, immunotherapy based on immune checkpoint inhibitors has achieved satisfactory results in the treatment of bladder cancer. However, this treatment is effective in some patients since other patients do not respond to this therapy [9]. Therefore, there is an urgent need to identify prognostic biomarkers for close monitoring of disease progress.

Considering the inherent biological heterogeneity of the tumor and the technical bias caused by sequencing platforms, the prognostic risk model should unify standardized gene expression profiles, a data analysis challenge. Some studies have explored the immune markers of bladder cancer patients used to predict the overall survival rate of the patients [10]. However, due to the inherent biological diversity of the tumor and the technical bias resulting from sequencing platforms, the clinical conversion of the prediction results is difficult, and the practicality is low [10]. Therefore, we analyzed and compared the matching values of the gene expression values to eliminate the inherent biological heterogeneity of the tumor and the technical bias caused by the sequencing platform. Based on the findings of previous research, this method is reliable, including in molecular cancer classification [11, 12].

Here, based on the immune-associated genes in the ImmPort database, we used the TCGA dataset and a microarray dataset GSE13507 [13, 14] to establish and verify signatures of 37 IRGPs targeting bladder cancer patients. Then, the risk score was used to predict the prognosis of bladder cancer. Finally, based on the 37 IRGPs, we divided bladder cancer patients into high- and low-risk score groups. We analyzed the differences between these two groups based on gene mutation, copy number variation, expression differences, and immune invasion to elucidate its potential pathogenic mechanism. This study provides prognostic biomarkers for patients with bladder cancer and provides a theoretical basis for close monitoring of disease progression and treatment stratification.

## 2. Materials and Methods

### 2.1. Data Downloading and Preprocessing

We retrieved the fragments per kilobase of transcript per million mapped reads (FPKM) standardized RNA sequencing dataset and clinical data from the TCGA repository (https://portal.gdc.cancer.gov/), containing 411 tumor samples and 19 nontumor samples. The gene expression value was converted to log2 (TPM + 1) for subsequent analysis. Then, we retrieved the gene expression data and clinical information in the GSE13507 dataset from the Gene Expression Comprehensive Database (https://www.ncbi.nlm.nih.gov/geo/), including primary tumor 165, normal 9, control 58, and recurrent 23. After normalization, we used the R package (org.Hs.eg.db) (TCGA) or GPL6102 platform annotation file (GSE13507) to convert the probes to gene names. If there was any gene with multiple probes, we selected the probe with the largest average expression value.

Additionally, we retrieved the masked somatic mutation (workflow type is VarScan2 variant aggregation and masking) and masked copy number segment data for bladder cancer patients from TCGA. SNP included 412 bladder patients, and CNV data included 414 tumor tissues and 394 normal tissues.

### 2.2. Construction of Signatures of Immune-Related Gene Pairs

We retrieved 1,811 immune-related genes (IRG) from the ImmPort database (https://immport.niaid.nih.gov) [15] for the construction of the immune-related prognostic signatures. There were 1,223 immune genes shared between the TCGA and GSE13507 datasets. Each IRGPs value was calculated by comparing the gene expression levels of specific samples in pairs. Specifically, in a pairwise comparison, if the first gene expression value was greater than the other gene expression value, the IRGPs were assigned a value of 1; otherwise, it was 0. We constructed 1495729 IRGPs.

### 2.3. Screening of IRGPs Related to Prognosis

The frequency of occurrence of each IRGP in the different tissues of TCGA and GSE13507 was counted. We used the Chi-square test to analyze the IRGPs that had differences in tumor tissues and normal tissues. *p* < 0.05 was used as the cutoff value for screening differential IRGPs. The IRGPs that showed differential expression in both the TCGA and GSE13507 datasets were selected as the differential IRGPs for further analysis.

We screened the bladder cancer patients with both survival time (>30 days) and survival status using the Cox proportional hazards regression model and survival analysis (log-rank test) to analyze the prognosis of differential IRGPs. We used *p* < 0.05 as the cutoff value for screening the prognostic-related IRGPs. We screened 251 prognostic-related IRGPs as the initial candidate IRGPs.

### 2.4. Construction of Prognostic Models of IRGPs

The patients in the TCGA dataset were randomly allocated to the TCGA-training set and the TCGA-testing set at a ratio of 7 : 3, and the ratio of each BC stage was the same. Using the initial candidate IRGPs, we conducted the least absolute shrinkage and selection operator (LASSO) regression analysis in the TCGA-training set. Subsequently, we calculated the individualized risk score using the coefficients and constructed a prognostic signature, which separates the high-risk score BC patients from the low-risk score group. The receiver operating characteristic (ROC) analysis was performed, and the area under the curve (AUC) was calculated at multiple time points to evaluate the discrimination.

Then, we verified the prognostic signature using the same coefficients and cutoff value in the TCGA-testing set and the external dataset GSE13507. At the same time, the prognostic model was presented as a risk map in each dataset, covering the expression level of the contained genes, the distribution of the risk scores, and the survival status of individuals.

### 2.5. Multivariate Cox Regression Analysis the Prognostic Value of Risk Score

We retrieved the clinical features from the TCGA and GSE13507 datasets, including survival time, survival status, age, gender, stage, lymph node metastasis, and distant metastasis status. We used the clinical data and risk scores for multiple cox regression analysis to determine the association linking the prognostic value of the risk scores and clinical characteristics. A value of *p* < 0.05 signified statistical significance.

### 2.6. SNP and CNV Analysis

We used the maftools R package [16] for visual analysis of gene mutations in the high- and low-risk score groups of BC.

When performing the CNV analysis, a value of segment mean less than −0.2 was recorded as −1 (i.e., missing copy number), greater than 0.2 was recorded as 1 (i.e., the copy number increased), and −0.2∼0.2 was recorded as 0 (i.e., wild type). We counted the frequency of the copy number variation in the high- and low-risk score groups and used the chi-square test in difference analysis.

### 2.7. Immune Cell Infiltration Analysis

We used the CIBERSORTx online tool (https://cibersortx.stanford.edu/index.php) [17] to analyze the degree of immune cell infiltration in the different samples. Gene expression data was used to estimate the gene expression profiles and provide abundance estimates of the member cell types in mixed cell populations. We used the RNA expression profile in the TCGA dataset for immune-infiltration analysis. Using the Monte Carlo sampling approach, CIBERSORT calculates the *p* value of the deconvolution for each sample to provide confidence in the estimation [17]. We selected the samples with *p* < 0.05 for subsequent analysis. We selected LM22 (22 immune cell types) as the signature matrix file, batch correction mode as B-mode, check bisable quantile normalization, run in absolute mode, and 1000 for permutations for significance analysis.

### 2.8. Gene Set Enrichment Analysis (GSEA)

We used the limma package for the high- and low-risk score group difference analysis. Bioconductor software package clusterProfiler was used for gene set enrichment analysis [18]. We reported the log2 fold change between the gene expression profiles of high- and low-risk groups. The gene sets from the high- vs. low-risk score groups were compared. The biological processes involved in this study were retrieved from the Molecular Signature Database (c2: curated gene sets, KEGG gene sets, gene symbols) (https://www.gsea-msigdb.org/gsea/downloads.jsp).

### 2.9. Statistical Analysis

All statistical tests were performed using R (R version 3.6.1, =http://www.r-project.org). For measurement data, we used the Wilcoxon test to compare the differences between groups. For counting data, the chi-square test or Fisher test was used to perform differential analysis. Cumulative survival time was calculated using the Kaplan–Meier method, and the differences in survival curves were analyzed using the log-rank test from the survival package. We performed univariate and multivariate analyses using the Cox proportional hazards regression model. We used the ggplot2 package to draw box and dumbbell diagrams and the heatmap package to draw heat maps. For all tests, a *p* < 0.05 indicated a statistically distinct difference. Statistical significance is shown as ^*∗*^*p* < 0.05,  ^*∗∗*^*p* < 0.01, and  ^*∗∗∗*^*p* < 0.001.

## 3. Results

### 3.1. Construction of Prognostic-Related IRGPs

**Figure 1:**
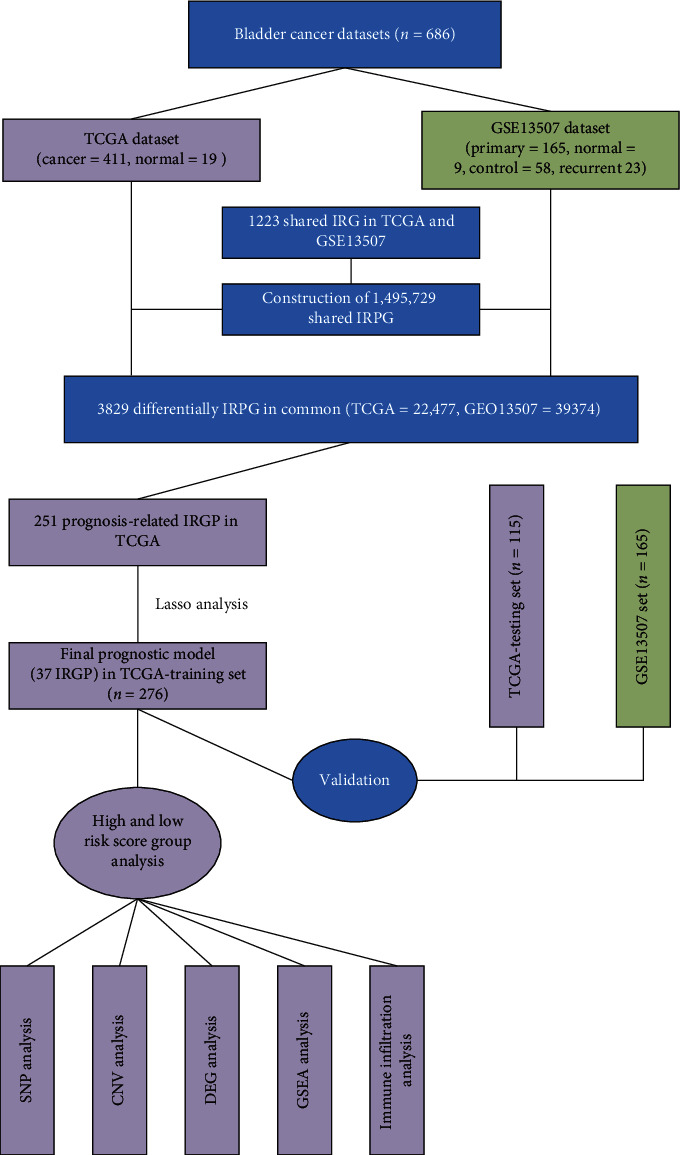
The process of model construction and subsequent analysis.

We retrieved 1,811 unique immune-related genes from the ImmPort database, of which 1,223 IRGs were shared between the TCGA and GSE13507 datasets. Then, we constructed 1495729 immune-related gene pairs (IRGPs) using unique 1223 immune genes. Moreover, we used the TCGA and GSE13507 datasets to construct differentially expressed IRGPs; TCGA included 430 tissues (411 tumor tissues and 19 normal tissues), and GSE13507 included 223 tissues (165 primary tumor tissues, 58 control groups). The fisher test results revealed that 22,477 and 39,374 IRGPs of TCGA and GSE13507, respectively, had differences. There were 3,829 differences in IRGPs between the two datasets. The analysis process can be seen in Figure 1.

We used the cox proportional hazards regression model and survival analysis (log-rank test) to screen the IRGPs related to prognosis among the differentially expressed IRGPs. Consequently, 391 bladder cancer patients were screened regarding survival status and survival time (>30 days) in the TCGA dataset, and 251 IRGPs were associated with prognosis (Supplementary Table 1). These 251 IRGPs related to prognosis were then utilized for subsequent model construction (Figure 1).

### 3.2. Development and Internal Validation of a Prognostic Signature

We recorded 391 BC patients in the TCGA dataset with a follow-up time of >30 days. The patients in the TCGA dataset were randomly assigned to the TCGA-training set (*n* = 276) and TCGA-testing set (*n* = 115) in 7 : 3 ratios based on the different stages. The clinical data of the patients in the TCGA-training set and TCGA-testing set groups are shown in Table 1. There was no statistical difference between the age, sex, stage, lymph node metastasis, and distant metastasis in the two groups of patients (Table 1, *p* > 0.05); therefore, further model construction could be conducted.

**Figure 2:**
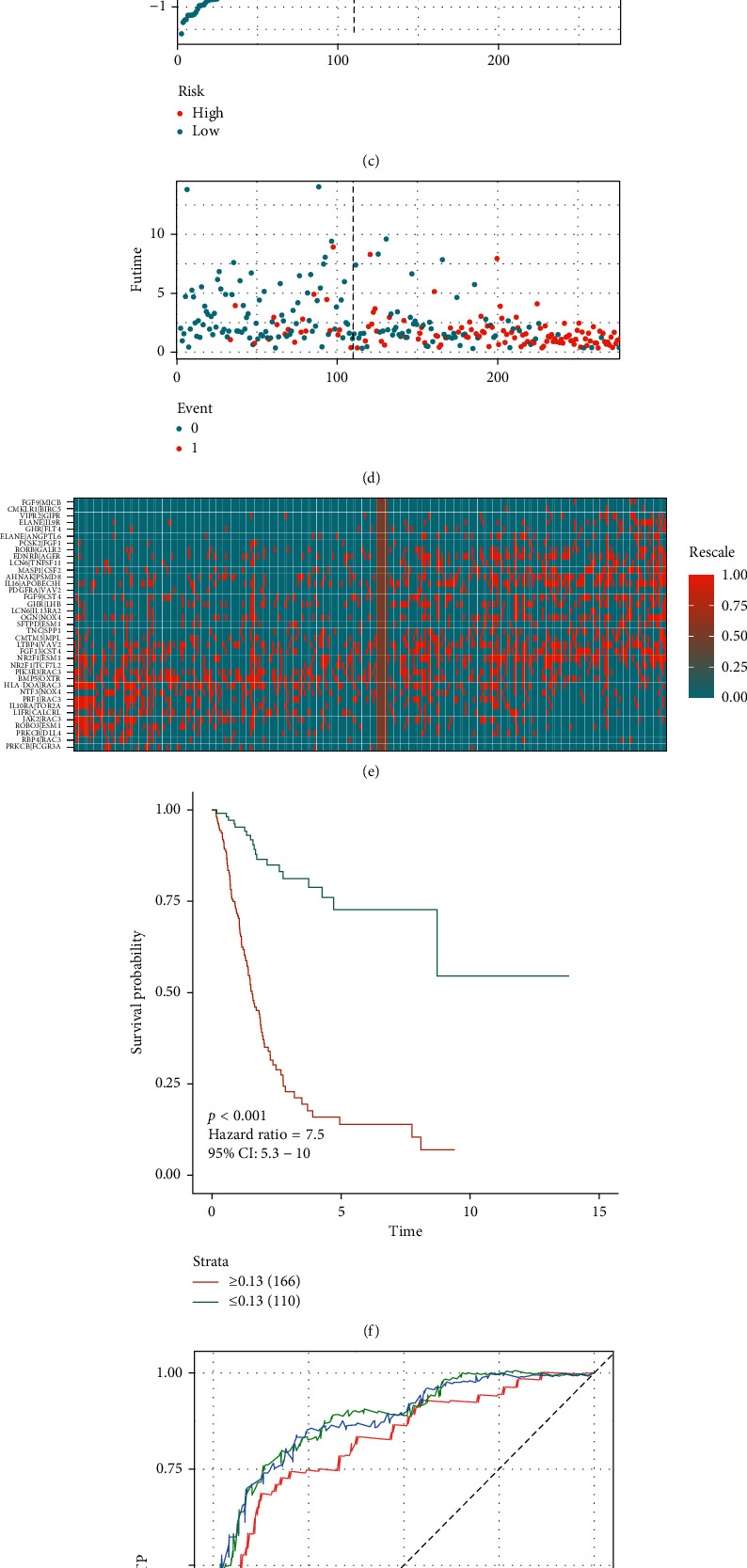
Development of the prognostic signature based on IRGPs in the TCGA-training set. (a, b) Identification of 37 IRGPs by LASSO regression analysis; (c) distribution of risk scores based on IRGPs in bladder patients; (d) survival status of patients in different groups; (e) heatmap of the expression profiles of IRGPs; (f) survival analysis for the signature-defined risk groups; (g) time-dependent ROC curve of the 37-IRGPs prognostic signature.

Subsequently, we defined the IRGPs coefficient (IRGPC) using the L1-penalized Cox proportional hazards regression on the TCGA-training set and selected 37 IRGPs in the final risk model (Table 2, Figures 2(a) and 2(b)). Then, we used IRGPC to calculate the risk score of each patient in the TCGA-training set and set the optimal cutoff value of IRGP for patients divided into high- or low-risk groups to −0.13 (Figure 2(c) and Supplementary Table 2). Figures 2(c)–2(e) shows the risk plot encompassing the distribution of the patients in the groups based on the signature, survival status of individuals between groups, and the expression level of included IRGPs. From this figure, there is a clear difference in the survival status between risk score groups, with the red dots signifying death and the blue dot survival. A significant number of deaths occurred in the high-risk score group. In contrast, a considerable number of patients in the low-risk score group survived throughout the follow-up. The risk score stratified the TCGA-training set remarkably and divided the patients into low- and high-risk score groups regarding the OS. Our data indicate that the OS of patients in the high-risk score group is distinctly lower compared with the low-risk score group (*p* < 0.001; HR = 7.5 [5.3, 10]) (Figure 2(f)). The ROC curve analysis (Figure 2(j)) revealed acceptable discrimination with AUCs of 0.820, 0.883, and 0.879 at 1-, 3-, and 5-year follow-up, respectively.

**Figure 3:**
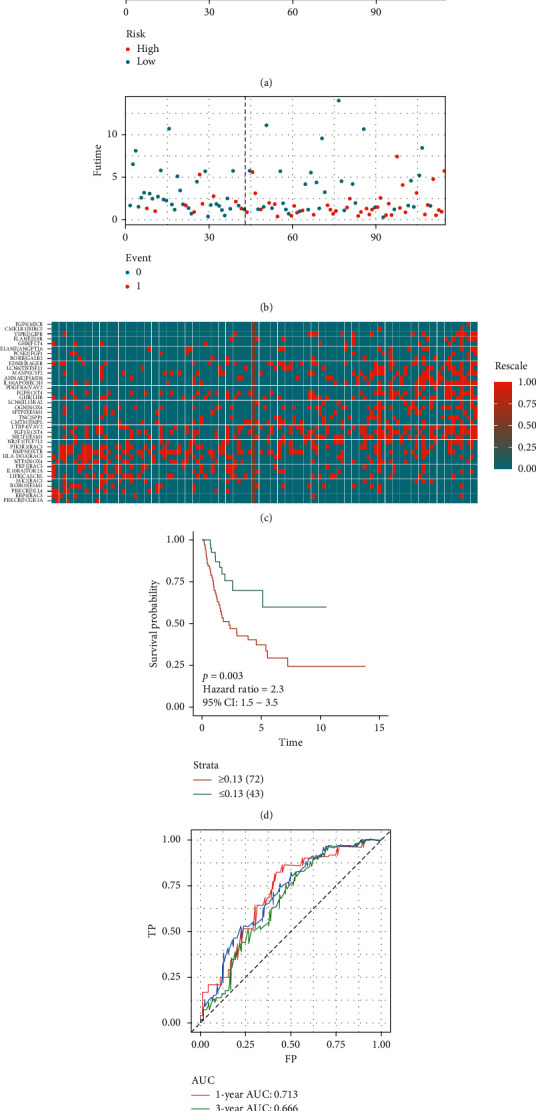
Development of a prognostic signature based on IRGPs in the TCGA-testing set. (a) Distribution of risk scores based on IRGPs in bladder patients; (b) survival status of patients in different groups; (c) heatmap of expression profiles of IRGPs; (d) survival analysis for the signature-defined risk groups; (e) time-dependent ROC curve of the 37-IRGPs prognostic signature.

We used IRGPC and cutoff values to evaluate the risk score of the BC patients in the TCGA-testing set. Consequently, the patients in the TCGA-testing set were divided into high- and low-risk score groups (see Supplementary Table 2 for specific scores and risk groups). Figures 3(a)–3(c) show the risk plot encompassing the distribution of the patients in the groups based on the signature, survival status of individuals between groups, and the expression level of included IRGPs. The figure reveals that a significant number of deaths occurred in the high-risk score group. However, a remarkable number of patients in the low-risk score group survived throughout the follow-up. The risk score stratified the TCGA-testing set significantly and divided the patients into low- and high-risk score groups based on the OS. Our data indicate that the OS of the patients in the high-risk score group is distinctly lower compared with the low-risk score group (*p*=0.003; HR = 2.3 [1.5, 3.5]) (Figure 3(d)). The ROC curve analysis (Figure 3(e)) revealed acceptable discrimination with AUCs of 0.713, 0.666, and 0.703 at 1-, 3-, and 5-year follow-up, respectively.

### 3.3. External Validation in GSE13507 Datasets

**Figure 4:**
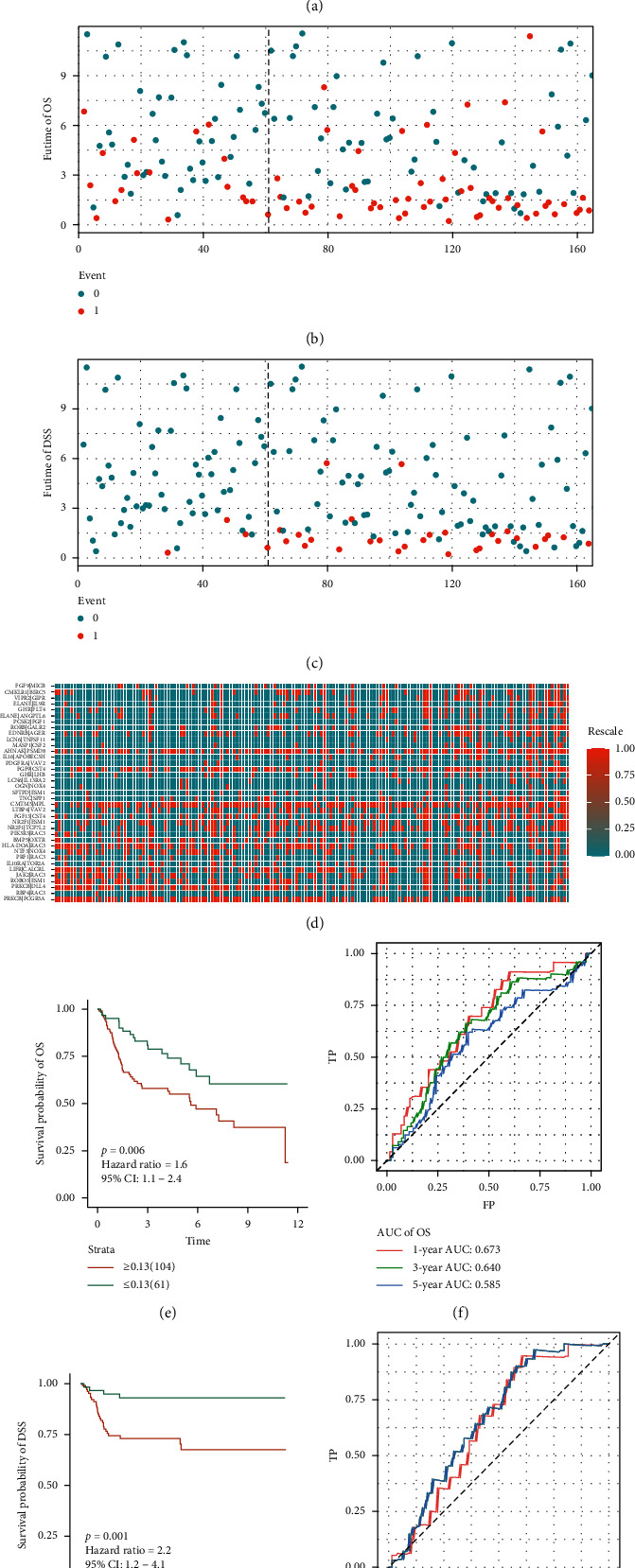
Development of the prognostic signature based on IRGPs in independent validation. GSE13507 dataset. (a) Distribution of risk scores based on IRGPs in bladder patients; (b) OS status of patients in different groups; (c) DSS status of patients in different groups; (d) heatmap of expression profiles of IRGPs; (e) survival analysis of OS between signature-defined risk groups; (f) time-dependent ROC curve of OS based on the 37-IRGPs prognostic signature; (g) survival analysis of DSS between signature-defined risk groups; (h) time-dependent ROC curve of DSS based on the 37-IRGPs prognostic signature.

In the GSE13507 cohort, we used the same IRGPC and cutoff values to calculate the risk score of each patient in the external validation GSE13507 and divided the patients into high- or low-risk score groups (see Supplementary Table 2 for specific scores and risk groupings). Figures 4(a)–4(d) shows the risk plot encompassing distribution of groups based on the signature, OS status of individuals between groups, disease-specific survival (DSS) status of individuals between groups, and the expression level of the included IRGPs. Similarly, a significant number of deaths occurred in high-risk score groups, whereas most of the patients in the low-risk score group remained alive during the follow-up. The risk scores stratified GSE13507 markedly and divided patients into the low- and high-risk score groups based on the OS and DSS. These results indicate that the OS of the patients in the high-risk score group is remarkably lower than in the low-risk score group (*p*=0.006; HR = 1.6 [1.1, 2.4]). Besides, we show that the DSS of the patients in the high-risk score group is markedly lower compared with the low-risk score group (*p*=0.001; HR = 2.2 [1.2, 4.1]) (Figures 4(e)–4(j)). The ROC curve analysis of OS (Figure 4(f)) revealed acceptable discrimination with AUCs of 0.673 and 0.640 at 1- , and 3-year follow-up, respectively. Moreover, the ROC curve analysis of DSS (Figure 4(h)) showed acceptable discrimination with AUCs of 0.644, 0.668, and 0.668 at 1-, 3-, and 5-year follow-up, respectively.

### 3.4. Validation of the Risk Score as an Independent Prognostic Factor

To further explore whether the risk score is an independent clinical prognostic factor, we performed univariate and multivariate Cox proportional hazards regression analysis on the TCGA and GSE13507 cohorts. In the TCGA cohort, the results of the single factor analysis indicated that the risk score, age, staging, lymph node metastasis, and distant metastasis are risk factors of OS in BC, and the multivariate analysis findings revealed that, after adjusting for age, lymph node metastasis, and distant metastasis, the risk score was still an independent prognostic factor for OS in BC (HR = 3.39 [2.65–4.34], *p* < 0.001) (Table 3). Similarly, in the GSE13507 cohort, our single factor analysis results posited that the risk score, age, lymph node metastasis, and distant metastasis are risk factors for OS in BC. The results of the multivariate analysis in the GSE13507 cohort revealed that after adjusting age, lymph node metastasis, and distant metastasis, the risk score is still a bladder cancer patient independent prognostic factor of OS (HR = 1.54 [1.01–2.35], *p*=0.046). Additionally, single-factor analysis posited that the risk score, age, staging, lymph node metastasis, and distant metastasis are risk factors for DSS in bladder cancer patients. However, the multifactorial analysis findings indicated that after adjusting age, staging, lymph node metastasis, and distant metastasis, risk core is not a bladder cancer independent prognostic factor for DSS (HR = 1.83 [1.00–3.36], *p*=0.0504).

### 3.5. Analysis of Gene Mutation, Variation in Copy Number, and Transcriptome Expression Differences in the Different Risk Score Groups

Our findings show that the risk score is an independent prognostic factor for bladder cancer patients and divides the patients into high- and low-risk score groups. Next, we investigated whether there are differences in gene mutation, variation in copy numbers, and transcriptome expression between the high- and low-risk groups.

**Figure 5:**
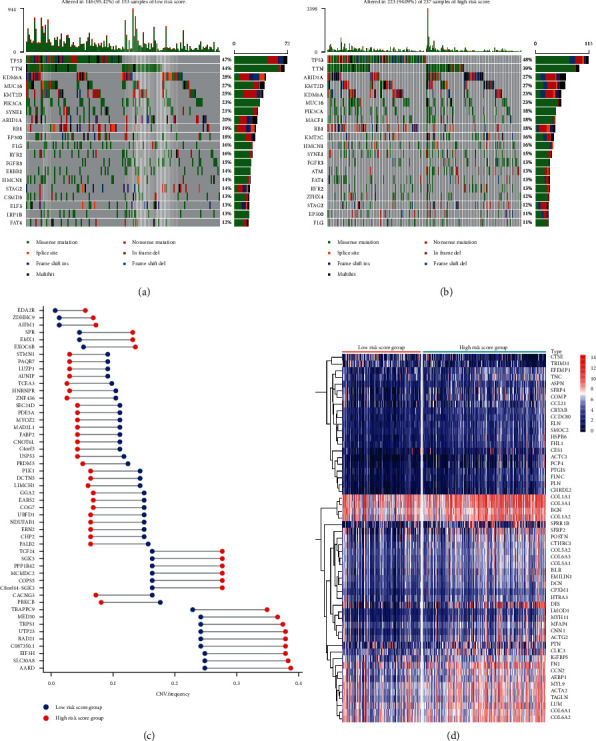
Analysis of gene mutation, copy number variation, and differential expression of the gene in different risk groups. (a) Visualization of gene mutations in patients of low-risk score groups; (b) visualization of gene mutations in patients of the high-risk score group; (c) dumbbell diagram showing copy number variation in different groups; (d) heatmap of differential expression of the gene in different groups.

Consequently, we found that the proportions of patients with gene mutations were 95.42% (146 in 153) and 94.09% (223 in 237) in the low-risk score and high-risk score groups, respectively (Figures 5(a) and 5(b)). The overall frequency of gene mutations is the same. However, there are significant differences in the frequency of single-gene mutations in different groups. In the top 20 gene mutation frequency, in the high-risk group, ARID1A (27% vs. 20%), MACF1 (18% vs. 9%), KMT2C (16% vs. 12%), etc. were remarkably higher compared with the low-risk score group. However, EP300 (11% vs. 18%), TTN (39% vs. 44%), KDM6A (23% vs. 28%), PIK3CA (18% vs. 23%), SYNE1 (15% vs. 21%), FLG (11% vs. 16%), ERBB2 (8% vs. 14%), LRP1B (7% vs. 13%), etc. were distinctly lower in the high-risk score group than in the low-risk score group (Supplementary Table 3).

In the high- and low-risk score group, the variation in gene copy numbers is significant (Figure 5(c)). Among the top 50 genes with different copy number variations, the frequency of copy number variation of EDA2R, ZDHHC9, AIFM1, SPR, EMX1, EXOC6B, TCG24, SGK3, PPP1R42, MCMDC2, COPS5, C8orf44-SGK3, TRAPPC9, MED30, TRPS1, UTP23, RAD21, C087350.1, EIF3H, SLC30A8, and AARD in the high-risk score group was markedly higher relative to the low-risk score group. However, the other genes were distinctly lower in the high-risk score group than in the low-risk score group. Notably, the copy number variation in the high- and low-risk score groups was primarily based on the copy number amplification (Supplementary Table 4). Besides, we identified five genes, namely ZNF436, TCEA3, HNRNPR, EMX1, and SPR, with the most significant differences in copy number variation. ZNF436, TCEA3, and HNRNPR have a high copy number variation frequency in the low-risk score group, and EMX1 and SPR have a high copy variation frequency in the high-risk score group.

We identified 55 differentially expressed genes in the high- and low-risk score groups (Figure 5(d) and Supplementary Table 5). In the high-risk score group, the expression levels of CTSE and TRIM31 were significantly lower relative to the low-risk score group, and the expression levels of the rest of the genes were higher compared with the low-risk score group.

### 3.6. Gene Set Enrichment Analysis

**Figure 6:**
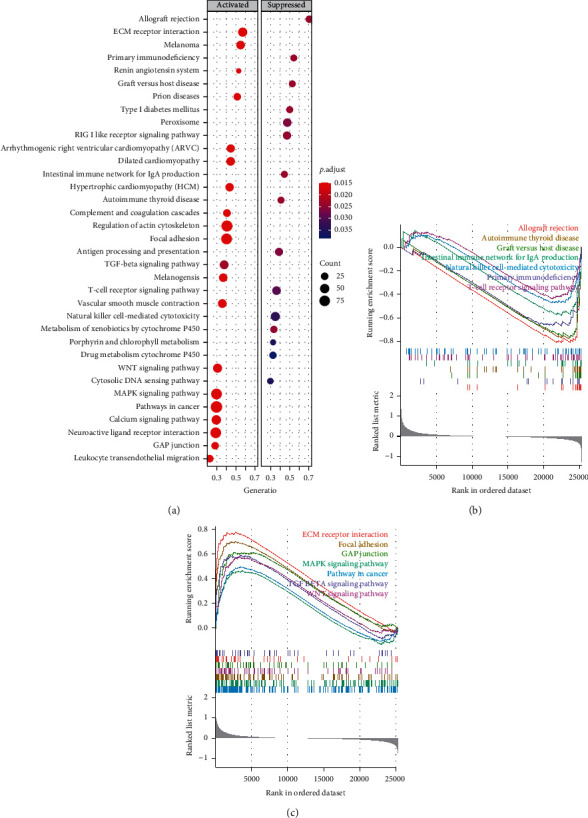
Gene set enrichment analysis of different groups. (a) Bubble chart of GSEA analysis in different groups; (b) GSEA analysis of immune-related pathways in different groups; (c) GSEA analysis of mesenchymal-related pathways in different groups.

To explore the underlying mechanism between the high- and low-risk score groups, we conducted the GSEA of the differential expression of high- and low-risk score groups. In the high-risk score group, various immune-related pathways were inhibited, including the allograft rejection, primary immunodeficiency, graft versus host disease, the intestinal immune network for IgA production, autoimmune thyroid disease, T-cell receptor signaling axis, and natural killer cell-mediated cytotoxicity pathways (Figures 6(a) and 6(b)). Additionally, in the high-risk score group, various mesenchymal phenotype-related pathways were activated, including the ECM receptor interaction, focal adhesion, TGF-*β* signaling axis, WNT signaling axis, MAPK signaling cascade, and the GAP junction (Figures 6(a) and 6(c)). Inhibition of the immune pathways and activation of mesenchymal phenotype-related pathways provide molecular mechanism evidence of bladder cancer prognosis, thereby predicting the prognosis of BC.

### 3.7. Difference of Tumor-Infiltrating Immune Cells between Different Risk Groups

**Figure 7:**
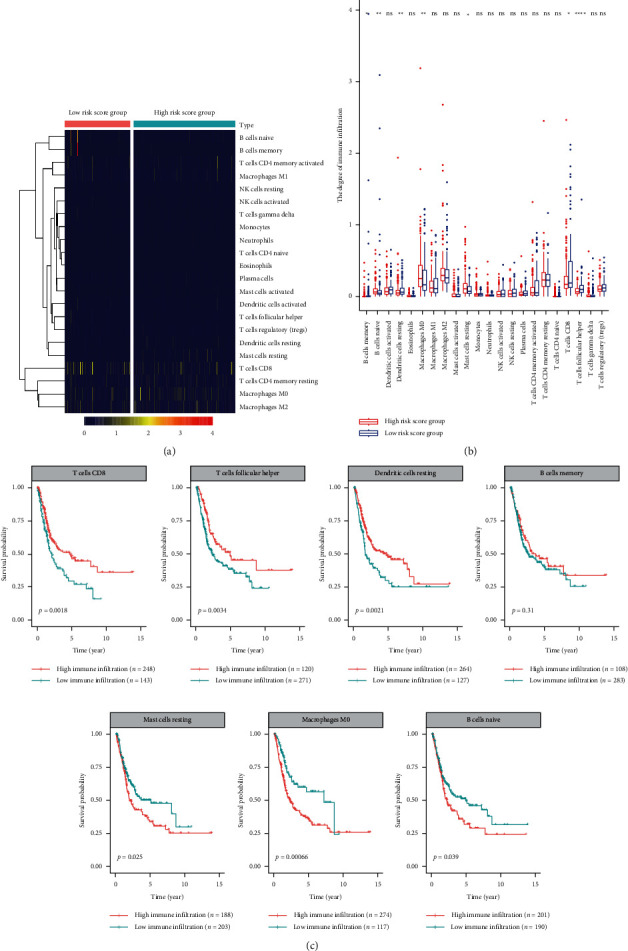
Analysis of immune infiltration in different groups. (a) Heatmap of infiltration of immune cells in different groups; (b) differential analysis of immune cells in different groups; (c) survival analysis of immune cells in BC.

Based on the GSEA analysis findings, the immune-related pathways are suppressed in the high-risk group. Therefore, we further explored the differences between immune cell infiltration in the high- and low-risk score groups. In the high-risk score group, the immune infiltration degree of memory B cells, resting dendritic cells, CD8 T cells, and follicular helper T cells was markedly lower than in the low-risk score group (Figures 7(a) and 7(b)). Moreover, the degree of immune infiltration of naïve B cells, M0 macrophages, and resting mast cells was significantly elevated in the high-risk score group. Subsequently, the survival analysis results showed that naïve B cells, M0 macrophages, and resting mast cells were associated with poor prognosis in BC patients (Figure 7(c)). The infiltrating immune cells, including memory B cells, resting dendritic cells, CD8 T cells, and follicular helper T cells, were associated with improved prognosis in BC patients (Figure 7(c)). Therefore, the degree of differential immune cell infiltration in the high- and low-risk score groups is strongly related to the prognosis of bladder cancer patients.

### 3.8. Differences in Immunoregulatory Gene Between the Different Risk Groups

**Figure 8:**
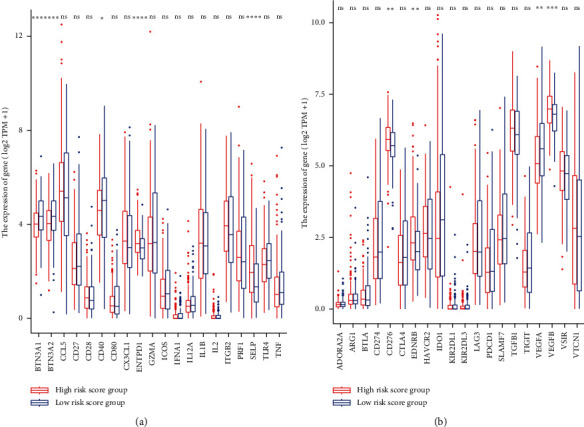
Differential expression of immunoregulatory genes in different groups. (a) Differential expression of immunostimulatory genes in different groups; (b) differential expression of immunosuppressive genes in different groups.

In the different risk score groups, the immune-regulatory genes were also partially different. Compared with the low-risk score group, some immunomodulatory genes, including BTN3A1, BTN3A2, and CD40, were markedly reduced in the high-risk score group (Figure 8(a)). However, ENTPD1 and SELP were remarkably elevated in the high-risk group compared with the low-risk group (Figure 8(a)). Compared with the low-risk score group, CD276, ENDRB, and VEGFB immune-suppressive genes were distinctly elevated in the high-risk score group. In contrast, VEGFA was remarkably reduced in the high-risk group relative to the low-risk group (Figure 8(b)). Therefore, the differential expression of some immunoregulatory genes constitutes a potential mechanism underlying the differences in the infiltration of different immune cells in different groups.

## 4. Discussion

Bladder cancer is the ninth most common malignant tumor in the world and ranks fifth in developed countries. At the initial hospital visit, approximately 20% of the patients are diagnosed with muscle infiltrative diseases. Due to disease recurrence, progression and high disease-specific mortality, multiple treatment modalities are required [19]. Localized bladder cancer is predominantly treated using chemotherapy, radiation therapy, and radical cystectomy, whereas patients with metastatic disease undergo systemic chemotherapy. Despite the aggressive treatment approach, there is still a poor prognosis in a considerable number of patients. The increase popularity of immunotherapy, including anti-PD1/PD-L1, and anti-CTLA-4 therapies, has shown great success in the treatment of human cancer, particularly in solid tumors. Cancers with a high mutation burden, including Hodgkin's lymphoma, melanoma, renal cell carcinoma, nonsmall cell lung cancer, and urinary tract epithelial bladder cancer, show promising response rates to anti-PD-1/PD-L1 antibody therapy [20–24]. However, most patients with BC respond poorly to immunotherapy. Therefore, there is a need for reliable immune-related biomarkers to stratify BC patients to identify patients with a high risk of recurrence and to guide adjuvant therapy.

To identify reliable prognostic biomarkers for bladder cancer, we preprocessed the TCGA data from the RNA-Seq platform and the GSE13507 data from the microarray-sequencing platform. Through the method of comparing values in the gene expression profile of a single sample, inherent technical differences on different platforms are reduced; therefore, there is no need to strictly and uniformly normalize the data. Hence, the 37 IRGPs could be potentially used for individualized and single-sample assessment of the survival of BC patients and can be easily applied in clinical practice.

Using the L1-penalized Cox proportional hazards regression on TCGA and GSE13507 data, we identified 37 IRGPs as potential clinical biomarkers for BC. Based on the prognostic characteristics of the 37 IRGPs, the overall survival rate and disease-specific survival rate of BC are divided into two subgroups with different survival outcomes, namely, the high- and low-risk score groups. The prognosis of the high-risk score group is significantly dismal compared with the low-risk score group. We validated our prognostic signature using the TCGA-testing set and GSE13507. The multivariate Cox analysis results showed that in TCGA, the IRGPs-based risk score is independent of clinical factors including age, stage, lymph node metastasis, and distant metastasis in bladder cancer patients, and the risk score is an independent prognostic marker. Similarly, in the GSE13507 cohort, we provided support that our risk score prognostic biosignature is independent for BC, implying that the prognostic characteristics based on 37 IRGPs are not related to the dataset.

To explore the mechanism of effectively stratifying BC patients based on the IRGPs signature, we divided the BC into high- and low-risk score groups based on the risk score. Next, we explored gene mutation, copy number variations, and gene expression differences between these groups. Consequently, our findings revealed no significant difference in the total frequency of gene mutations between the two groups. However, there was a remarkable difference in the frequency of single-gene mutations between the two groups. For instance, in the high-risk score group, the mutation frequency of lysine-specific methyltransferase 2C (KMT2C) was higher. KMT2C is a tumor suppressor, and cells with low KMT2C activity lack homologous recombination-mediated double-strand break DNA repair, resulting in a significantly high rate of endogenous DNA damage and genomic instability [25]. Additionally, the findings of studies show that specific mutations in EP300 predict a lower risk of recurrence and reduce breast cancer mortality [26]. In the high-risk score group, the mutation frequency of EP300 is higher. In BC, the EP300 mutations are associated with a higher burden of tumor mutations and indicate a good clinical prognosis [27]. The differences in different gene mutations could be a potential mechanism for poor prognosis in the high-risk score groups.

Regarding copy number variation, we established that in both the high- and low-risk score groups, copy number variation is primarily based on copy number amplification. We identified five genes, namely ZNF436, TCEA3, HNRNPR, EMX1, and SPR, with the most significant differences in copy number variation. ZNF436, TCEA3, and HNRNPR had a high copy number variation frequency in the low-risk score group, whereas EMX1 and SPR had a high copy variation frequency in the high-risk score group. ZNF436 is a member of the zinc finger transcription factor family and acts as a negative modulator in gene transcription mediated by the MAPK signaling pathway [28]. In ovarian cancer cells, the interaction between TCEA3 and TGF*β* receptor-I is activated through the Smad-dependent JNK pathway, which induces the death of ovarian cancer cells [29]. SPR and its downstream metabolite BH4 are critical modulators of T cell biology and are easily manipulated to modulate anticancer immunity [30]. The differences in copy number variations affect the prognosis of bladder cancer patients.

At the same time, the transcriptome levels in BC patients also differed in the high- and low-risk score groups. The CRYAB gene had the most significant difference in expression levels in the high- and low-risk score groups. As an antiapoptotic protein, CRYAB negatively regulates the apoptotic members of the Bcl-2 family, Bax, and caspase-3 through multiple signaling pathways [31, 32]. Additionally, intracellular CRYAB is an effective factor in controlling neuroinflammation in various situations [33]. Macrophages promote the metastasis of non-small cell lung cancer by upregulating CRYAB [34]. Some other differential genes are also related to immune modulation. Therefore, we conducted GSEA analysis and found that in the high-risk score group, multiple immune-related signaling pathways are inhibited, including T cell receptor signaling pathway and natural killer cell-mediated cytotoxicity cascade. Moreover, we established that the mesenchymal-related signaling pathways consisting of the ECM receptor interaction, focal adhesion, TGF-*β* signaling pathway, WNT signaling pathway, MAPK signaling pathway, and GAP junction are activated. Therefore, in BC, gene mutations, copy number variations, and differential transcriptome expression affect the poor prognosis of high-risk score patients through immunosuppression and matrix activation.

To further verify the mechanism of immunity in the high- and low-risk score BC patients, we analyzed the differences in the degree of immune cell infiltration and immune-regulatory genes in the groups. We established that the naïve B cells, M0 macrophages, and resting mast cells are significantly elevated in the high-risk score group, which is related to the poor prognosis of bladder cancer patients. Besides, we found that the memory B cells, resting dendritic cells, CD8 T cells, and follicular helper T cells are significantly reduced in the high-risk score group, and their low infiltration level is associated with poor prognosis in patients with BC. CD8+ T lymphocytes are major antitumor effector cells [35]. Increasing research evidence shows that CD8 T cells mediate the regression of various tumors, resulting in durable long-term disease remissions [36]. T follicular helper (Tfh) cells have protective roles in nonlymphoid tumors. Higher levels of Tfh cells infiltrate, and their ability to organize tertiary lymphoid structures within tumors is associated with increased survival and reduced immunosuppression, which strongly correlate with increased survival in breast cancer [37]. Here, the immunosuppressive genes (CD276, ENDRB, and VEGFB) were significantly elevated in the high-risk score group. CD276 is a member of the immune-modulator family and coordinates antitumor immunity. The overexpression of CD276 is related to poor prognosis in tumor patients and the potential of tumor invasion and metastasis [38, 39]. In conclusion, the differences in the tumor immune microenvironment (including immune cell infiltration and immune-regulatory genes) are potentially responsible for the differences in survival outcomes observed between the two groups defined by prognostic markers.

Herein, we report a prognostic signature based on IRGPs to predict the survival rate of BC patients, which has clinical significance and effectiveness in different datasets. To the best of our knowledge, this is the first IRGPs-based signature reported in BC. However, our study did not entail in vitro and in vivo experiments; therefore, the results cannot fully elucidate the molecular mechanism of BC. Therefore, it is necessary to conduct further research.

## 5. Conclusions

We systematically studied the prognostic value of 37 IRGPs as potential independent prognostic factors for BC and provided a risk assessment for the prognosis of BC. In the high- and low-risk score patients, there are differences in gene mutations, copy number variations, and gene expression levels. The changes in immune-related signaling pathways and mesenchymal-related signaling pathways in the two groups affect the prognosis of patients with BC. The biosignatures based on 37 IRGPs are promising for BC prognosis.

## Figures and Tables

**Table 1:** Clinical characteristics of bladder cancer patients in different datasets.

Characteristic	TCGA cohort			Validation cohort
	Training set	Testing set	*p* value	GSE13507
No. of samples	276	115		165

Median age in years (range)	68 (34–88)	69 (37–89)	0.8731	66 (24–88)

Gender				
Male	207	83	0.6492	135
Female	69	32		30

Stage				
T0	1	0	0.9500	24
T1	3	0		80
T2	96	41		31
T3	133	56		19
T4	42	18		11
TX	1	0		0

Lymph node metastasis				
N0	153	72	0.8184	149
N1	34	10		8
N2	54	21		6
N3	6	1		1
NX	26	10		1
NA	3	1		0

Distant metastasis				
M0	130	57	0.7559	158
M1	8	2		7
MX	137	55		0
NA	1	1		0

**Table 2:** Model information about IRGPs.

IRG 1	Full name	IRG 2	Full name	Lasso coefficient
FGF9	Fibroblast growth factor 9	MICB	MHC class I polypeptide-related sequence B	0.2288
CMKLR1	Chemokine-like receptor 1	BIRC5	Baculoviral IAP repeat-containing 5	0.1965
VIPR2	Vasoactive intestinal peptide receptor 2	GIPR	Gastric inhibitory polypeptide receptor	0.3493
ELANE	Elastase, neutrophil expressed	IL9R	Interleukin 9 receptor	0.2896
GHR	Growth hormone receptor	FLT4	Fms-related tyrosine kinase 4	0.0652
ELANE	Elastase, neutrophil expressed	ANGPTL6	Angiopoietin-like 6	0.0617
PCSK2	Proprotein convertase subtilisin/kexin type 2	FGF1	Fibroblast growth factor 1	0.0177
RORB	RAR-related orphan receptor B	GALR2	Galanin receptor 2	0.2577
EDNRB	Endothelin receptor type B	AGER	Advanced glycosylation end product-specific receptor	0.0772
LCN6	Lipocalin 6	TNFSF11	Tumor necrosis factor superfamily, member 11	0.1041
MASP1	Mannan-binding lectin serine peptidase 1	CSF2	Colony stimulating factor 2	0.1981
AHNAK	AHNAK nucleoprotein	PSMD8	Proteasome 26S subunit, non-ATPase, 8	0.2832
IL16	Interleukin 16	APOBEC3H	Apolipoprotein B mRNA editing enzyme, catalytic polypeptide-like 3H	0.0421
PDGFRA	Platelet-derived growth factor receptor, alpha polypeptide	VAV2	VAV 2 guanine nucleotide exchange factor	0.0141
FGF9	Fibroblast growth factor 9	CST4	Cystatin S	0.0840
GHR	Growth hormone receptor	LHB	Luteinizing hormone beta polypeptide	0.0237
LCN6	Lipocalin 6	IL13RA2	Interleukin 13 receptor, alpha 2	0.3899
OGN	Osteoglycin	NOX4	NADPH oxidase 4	0.0529
SFTPD	Surfactant protein D	ESM1	Endothelial cell-specific molecule 1	0.3064
TNC	Tenascin C	SPP1	Secreted phosphoprotein 1	0.2258
CMTM5	CKLF-like MARVEL transmembrane domain containing 5	MPL	Myeloproliferative leukemia virus oncogene	0.1306
LTBP4	Latent transforming growth factor beta binding protein 4	VAV2	VAV 2 guanine nucleotide exchange factor	0.1395
FGF13	Fibroblast growth factor 13	CST4	Cystatin S	0.0819
NR2F1	Nuclear receptor subfamily 2, group F, member 1	ESM1	Endothelial cell-specific molecule 1	0.0037
NR2F1	Nuclear receptor subfamily 2, group F, member 1	TCF7L2	Transcription factor 7-like 2	0.1656
PIK3R3	Phosphoinositide-3-kinase, regulatory subunit 3 (gamma)	RAC3	Ras-related C3 botulinum toxin substrate 3	−0.1612
BMP5	Bone morphogenetic protein 5	OXTR	Oxytocin receptor	−0.2999
HLA-DOA	Major histocompatibility complex, class II, DO alpha	RAC3	Ras-related C3 botulinum toxin substrate 3	−0.1058
NTF3	Neurotrophin 3	NOX4	NADPH oxidase 4	−0.1384
PRF1	Perforin 1	RAC3	Ras-related C3 botulinum toxin substrate 3	−0.1295
IL10RA	Interleukin 10 receptor, alpha	TOR2A	Torsin family 2, member A	−0.1601
LIFR	Leukemia inhibitory factor receptor alpha	CALCRL	Calcitonin receptor-like	−0.2448
JAK2	Janus kinase 2	RAC3	Ras-related C3 botulinum toxin substrate 3	−0.3199
ROBO3	Roundabout, axon guidance receptor, homolog 3	ESM1	Endothelial cell-specific molecule 1	−0.6215
PRKCB	Protein kinase C, beta	DLL4	Delta-like 4	−0.2046
RBP4	Retinol binding protein 4, plasma	RAC3	Ras-related C3 botulinum toxin substrate 3	−0.5189
PRKCB	Protein kinase C, beta	FCGR3A	Fc fragment of IgG, low affinity IIIa, receptor	−0.2420

**Table 3:** Univariate and multivariate analyses of prognostic factors in the TCGA data set and independent validation data set.

Characteristics	Univariate			Multivariate		
	Hazard ratio	CI95	*p* value	Hazard ratio	CI95	*p* value
TCGA dataset (OS)						
Riskscore	3.95	3.12–4.99	<0.0001	3.39	2.65–4.34	<0.0001
Age	1.85	1.25–2.73	0.0020	1.5	1.01–2.22	0.0452
Gender	1.19	0.86–1.66	0.2932	NA	NA	NA
Stage_T	1.69	1.37–2.09	0.0360	1.35	1.07–1.71	0.0104
Stage_N	1.15	1.05–1.25	0.0015	1.18	1.07–1.3	0.0011
Stage_M	1.11	1.01–1.23	<0.0001	1.01	0.91–1.13	0.7855

GSE13507 dataset (OS)						
Riskscore	1.58	1.05–2.37	0.0270	1.54	1.01–2.35	0.0462
Age	3.9	1.86–8.19	0.0003	3.62	1.72–7.62	0.0007
Gender	1.56	0.88–2.77	0.1290	NA	NA	NA
Stage_T	1.03	0.89–1.2	0.7038	NA	NA	NA
Stage_N	2.71	1.99–3.69	<0.0001	2.19	1.52–3.13	<0.0001
Stage_M	9.9	4.38–22.37	<0.0001	2.62	1.05–6.54	0.0397

GSE13507 dataset (DSS)						
Riskscore	2.24	1.23–4.08	0.0087	1.83	1–3.36	0.0504
Age	3.12	1.09–8.9	0.0336	3.18	1.08–9.34	0.0355
Gender	2.1	0.97–4.54	0.0600	NA	NA	NA
Stage_T	1.34	1.09–1.63	0.0046	1.34	1.06–1.7	0.0155
Stage_N	3.29	2.36–4.58	<0.0001	2.55	1.77–3.69	<0.0001
Stage_M	13.12	5.28–32.6	<0.0001	2.52	0.92–6.85	0.0708

## Data Availability

The datasets used and analyzed during the current study are available from the corresponding author on reasonable request.

## Supplementary Materials

Supplementary Materials Supplementary Table 1: 251 IRGPs related to prognosis. Supplementary Table 2: risk score of bladder cancer patients in TCGA dataset and GSE13507 dataset. Supplementary Table 3: mutation frequency of some genes in different risk score groups. Supplementary Table 4: copy number variation of the top 50 genes in different risk score groups. Supplementary Table 5: differentially expressed genes in different risk score groups.
