# Supplementary material for: Prognostic Implications of Immune-Related Gene Pairs Signatures in Bladder Cancer
Source: J Oncol. 2021 Jul 26;2021:5345181. doi: 10.1155/2021/5345181 (PMC8331311; doi:10.1155/2021/5345181)
Supplement: Supplementary Materials — Supplementary Table 1: 251 IRGPs related to prognosis. Supplementary Table 2: risk score of bladder cancer patients in TCGA dataset and GSE13507 dataset. Supplementary Table 3: mutation frequency of some genes in different risk score groups. Supplementary Table 4: copy number variation of the top 50 genes in different risk score groups. Supplementary Table 5: differentially expressed genes in different risk score groups. [file 5345181.f1.zip › 5345181.f1/Supplementary tables 1 (2).pdf]

IRGPs	Cox proportional hazards regression model			Survival analysis (Log-Rank test)
	HR	95% CI	p.value	pValue_log
A2M|ADRM1	1.4	(1-2)	0.025	0.023848052
A2M|PPP4C	1.4	(1-1.9)	0.038	0.036985458
AHNAK|PSMD8	1.7	(1.3-2.4)	0.00069	0.000591918
BMP5|IL9R	1.5	(1.1-2.1)	0.021	0.020204629
BMP5|OXTR	0.72	(0.52-1)	0.048	0.046626636
CD4|SPP1	0.57	(0.35-0.93)	0.025	0.023508937
CD40|SPP1	0.68	(0.47-0.98)	0.041	0.039471066
CMKLR1|BIRC5	3.4	(1.4-8.2)	0.0078	0.004679801
CMTM5|KLRK1	1.9	(1.3-2.7)	0.00039	0.000306166
CMTM5|MPL	1.6	(1.2-2.3)	0.0048	0.004373666
COLEC12|BID	1.9	(1-3.4)	0.047	0.043176709
COLEC12|CCR1	1.5	(1.1-2.1)	0.012	0.011302828
COLEC12|DCK	1.7	(1.1-2.7)	0.021	0.019323914
COLEC12|PLXNC1	1.4	(1-1.9)	0.028	0.027197313
CTSG|IL17RB	1.4	(1-2)	0.027	0.02580206
CTSG|TMSB15A	1.5	(1.1-2)	0.015	0.014502464
CXCL12|CRLF3	1.4	(1-1.9)	0.049	0.04771495
CXCL12|DHX58	1.5	(1.1-2.1)	0.013	0.012537363
CXCL12|IKBKE	1.5	(1.1-2.1)	0.012	0.011276647
CXCL12|IL17RA	1.4	(1-1.9)	0.032	0.030710533
CXCL12|IRF5	1.6	(1.2-2.2)	0.0046	0.004254389
CXCL12|KRAS	1.5	(1-2)	0.026	0.025166075
CXCL12|MAP2K2	2	(1-3.9)	0.043	0.039031115
CXCL12|MX2	1.4	(1-1.9)	0.023	0.022076643
CXCL12|NFKBIE	1.6	(1-2.4)	0.032	0.029954869
CXCL12|RELB	1.6	(1.1-2.4)	0.013	0.011940116
CXCL12|SH3BP2	1.6	(1.2-2.1)	0.0039	0.003533096
CXCL12|TCF7L2	1.4	(1-1.9)	0.039	0.038147332
CXCL12|TOR2A	1.5	(1.1-2.1)	0.0055	0.005047921
DES|APOBEC3C	1.4	(1-1.9)	0.025	0.024480598
DES|CDH1	1.5	(1.1-2)	0.017	0.016083136
DES|FURIN	1.4	(1.1-1.9)	0.018	0.016909037
DES|HSPA4	1.4	(1-1.9)	0.034	0.033171605
DES|MAP2K2	1.4	(1.1-1.9)	0.018	0.017691689
DES|MDK	1.5	(1.1-2.1)	0.0084	0.007931922
DES|NENF	1.4	(1-1.9)	0.043	0.041779533
DES|PDIA3	1.5	(1-2.1)	0.025	0.02443067
DES|PLAU	1.4	(1-1.9)	0.045	0.044388174
DES|PLTP	1.6	(1.2-2.1)	0.0032	0.00297706
DES|PSMD13	1.5	(1.1-2)	0.0065	0.006129249
DES|PSMD3	1.4	(1-1.9)	0.025	0.024323728
DES|SPP1	1.4	(1.1-1.9)	0.019	0.018329039
DES|SRC	1.5	(1.1-2)	0.015	0.014288844
DES|TGFB1	1.4	(1.1-1.9)	0.017	0.016666456
DES|TINAGL1	1.6	(1.2-2.1)	0.004	0.003705479
DES|TRPC4AP	1.4	(1.1-1.9)	0.02	0.019690686
EDNRA|CACYBP	2.5	(1-6.2)	0.041	0.034009002
EDNRA|ELAVL1	2.8	(1.2-6.3)	0.013	0.009745408
EDNRA|GSK3B	1.7	(1-2.8)	0.035	0.033156019
EDNRA|HMOX1	3.1	(1.3-7.7)	0.013	0.008933792
EDNRA|NRAS	3.1	(1.4-7.1)	0.0062	0.003818873
EDNRA|PLXNA3	1.6	(1.1-2.2)	0.0062	0.005735176
EDNRA|PTPN6	2.3	(1.3-4.1)	0.0054	0.004208861
EDNRA|TRIM27	1.7	(1-2.7)	0.04	0.037807281
EDNRA|UNC93B1	2.7	(1.5-5.1)	0.0014	0.000845234
EDNRA|VAV2	1.9	(1.3-2.9)	0.0019	0.00153321

EDNRB|AGER	1.8 (1.3-2.5)	0.00018	0.000141541
ELANE|ANGPTL6	1.8 (1.2-2.8)	0.0042	0.003629238
ELANE|BIRC5	27 (3.5-200)	0.0015	1.53E-06
ELANE|CCR10	1.6 (1-2.6)	0.043	0.041227023
ELANE|CD244	1.6 (1.1-2.4)	0.018	0.016507664
ELANE|CSF2	1.6 (1.1-2.3)	0.011	0.009906392
ELANE|CST4	1.7 (1.2-2.3)	0.0011	0.000943274
ELANE|GALR2	1.5 (1-2.2)	0.04	0.038115594
ELANE|GIPR	2 (1.2-3.3)	0.0044	0.003654019
ELANE|HAMP	1.4 (1-2)	0.043	0.041869207
ELANE|IL9R	2.1 (1.4-3.3)	0.00055	0.000403675
ELANE|INSL3	1.8 (1.1-2.9)	0.026	0.023615656
ELANE|KLRC1	1.6 (1.2-2.2)	0.0045	0.004117513
ELANE|LAT	1.5 (1-2.1)	0.036	0.035013526
ELANE|VGF	2 (1.2-3.4)	0.0083	0.007050401
FCGR2B|BIRC5	14 (1.9-110)	0.01	0.000672953
FGF10|CSPG5	1.5 (1.1-2.1)	0.023	0.022210236
FGF10|VGF	1.5 (1-2.1)	0.041	0.039554899
FGF13|CST4	1.6 (1.2-2.2)	0.0025	0.00231951
FGF13|FGF1	0.55 (0.31-0.97)	0.037	0.034608046
FGF9|APOBEC3H	2.4 (1.3-4.6)	0.0079	0.006151209
FGF9|CCL17	2.5 (1.2-5.3)	0.019	0.015220429
FGF9|CD72	3.1 (1.4-7)	0.0072	0.004663022
FGF9|CSF2	1.7 (1.2-2.5)	0.0031	0.00272263
FGF9|CST4	1.9 (1.4-2.6)	0.00011	8.77E-05
FGF9|GHRL	1.8 (1.1-2.7)	0.012	0.011043996
FGF9|IL1RAP	2.4 (1.1-5.2)	0.022	0.017900574
FGF9|IL20RB	1.9 (1-3.7)	0.044	0.040441024
FGF9|IL2RA	2.4 (1.1-5.6)	0.033	0.02731436
FGF9|INSL3	1.8 (1.1-3.2)	0.029	0.026476133
FGF9|MICB	4.8 (1.8-13)	0.0022	0.000731935
FGF9|NOX1	2 (1-3.9)	0.045	0.040484284
FGF9|PDK1	3.5 (1.1-11)	0.034	0.023974718
FGF9|PROC	1.6 (1-2.6)	0.037	0.035325159
FGF9|RASGRP3	2.3 (1.1-4.9)	0.033	0.027827625
FGF9|RLN1	1.4 (1-2)	0.046	0.044735632
FGF9|SEMA4G	2.5 (1.1-5.6)	0.029	0.023730578
FGF9|TFR2	2.3 (1.3-4.1)	0.0062	0.004882987
FGF9|ULBP2	2.1 (1-4.6)	0.049	0.043940642
FGF9|WFIKKN1	1.5 (1.1-2.1)	0.015	0.013970083
GHR|APOM	1.8 (1.1-3)	0.031	0.029115033
GHR|C8G	1.7 (1.2-2.4)	0.0036	0.003286807
GHR|FLT3LG	1.6 (1.1-2.4)	0.027	0.025881009
GHR|FLT4	2.3 (1.4-3.6)	7.00E-04	0.000498358
GHR|GNRH1	1.5 (1.1-2.1)	0.019	0.018505629
GHR|HSPA1L	1.6 (1.1-2.4)	0.014	0.01293996
GHR|IL17RB	1.6 (1-2.6)	0.034	0.032334809
GHR|LHB	1.5 (1.1-2.1)	0.011	0.010208742
GHR|MICB	1.9 (1.1-3.4)	0.033	0.030648203
GHR|NPFF	1.6 (1.2-2.2)	0.005	0.004640979
GHR|NUDT6	1.6 (1.2-2.2)	0.0027	0.002437628
GHR|PDK1	1.9 (1.2-3)	0.0051	0.004407898
GHR|RASGRP3	2 (1.4-3)	0.00051	0.000394819
GHR|TFR2	1.5 (1.1-2)	0.023	0.021762608
GHR|TMSB15A	1.7 (1.2-2.6)	0.0065	0.005878804
GHR|TNFRSF10C	1.6 (1.1-2.2)	0.0069	0.00647831
GHR|TNFRSF4	1.9 (1-3.5)	0.039	0.03577974
GHR|UCN	1.6 (1.1-2.3)	0.013	0.01253951

GNAI1|SPP1	0.65 (0.44-0.97)	0.034	0.032651885
GREM2|PROC	1.4 (1-1.9)	0.043	0.042109063
HLA-DOA|RAC3	0.67 (0.48-0.93)	0.015	0.014838876
HLA-DOA|SPP1	0.54 (0.31-0.94)	0.029	0.026434741
IL10RA|TOR2A	0.63 (0.41-0.97)	0.038	0.036192179
IL16|APOBEC3H	2 (1.5-2.7)	9.50E-06	6.26E-06
IL16|INHBA	0.56 (0.33-0.95)	0.03	0.027988089
IL33|MX2	1.4 (1-1.9)	0.028	0.027159154
JAK2|RAC3	0.57 (0.37-0.89)	0.014	0.013145302
LCN6|BIRC5	27 (3.5-200)	0.0015	1.53E-06
LCN6|CST4	1.5 (1.1-2)	0.016	0.015440342
LCN6|IL13RA2	1.7 (1-2.8)	0.034	0.031882117
LCN6|TNFSF11	1.6 (1.1-2.4)	0.021	0.019610243
LIFR|CALCRL	0.58 (0.38-0.89)	0.012	0.010551976
LTBP4|CMTM7	1.5 (1.1-2.1)	0.0073	0.00694054
LTBP4|OGFR	1.5 (1.1-2.2)	0.019	0.01803514
LTBP4|PLXNB1	1.6 (1.1-2.2)	0.0063	0.005918586
LTBP4|TINAGL1	1.8 (1.1-3)	0.015	0.013391148
LTBP4|TYK2	1.5 (1.1-2)	0.016	0.015314507
LTBP4|VAV2	1.6 (1.2-2.1)	0.0036	0.003402916
MASP1|CSF2	1.6 (1.1-2.2)	0.01	0.00987557
MASP1|CST4	1.5 (1.1-2)	0.016	0.015546102
NGF|BIRC5	27 (3.5-200)	0.0015	1.53E-06
NGF|IL17RB	1.5 (1.1-2.1)	0.011	0.010135234
NR2F1|BID	2 (1.2-3.3)	0.0064	0.005379512
NR2F1|BRAF	1.6 (1.2-2.1)	0.0038	0.00355477
NR2F1|DHX58	1.4 (1-2.1)	0.045	0.043783512
NR2F1|ESM1	1.6 (1.2-2.1)	0.0034	0.003112993
NR2F1|GSK3B	1.5 (1-2.2)	0.047	0.04530674
NR2F1|IFNAR2	1.5 (1.1-2.1)	0.0066	0.006202244
NR2F1|NR2C1	1.5 (1.1-2)	0.0085	0.007996803
NR2F1|PLXNA3	1.6 (1.1-2.2)	0.0055	0.005064625
NR2F1|TANK	1.6 (1.1-2.2)	0.013	0.011939982
NR2F1|TCF7L2	1.5 (1.1-2.1)	0.012	0.011499906
NR2F1|TRIM27	1.8 (1.2-2.8)	0.0068	0.005989286
NR2F1|VAV2	1.5 (1-2.2)	0.038	0.037291806
NR3C2|ESM1	1.5 (1-2.2)	0.039	0.038124822
NTF3|NOX4	0.61 (0.4-0.92)	0.02	0.018370636
NTF3|TUBB3	0.51 (0.3-0.85)	0.01	0.008800271
OGN|FGF1	1.4 (1.1-1.9)	0.017	0.016722793
OGN|NOX4	1.6 (1.2-2.2)	0.0015	0.00130932
PAK3|CST4	1.4 (1-1.9)	0.046	0.045239532
PCSK2|CGB8	1.4 (1-1.9)	0.035	0.034475723
PCSK2|CST4	1.5 (1.1-2.1)	0.022	0.021508246
PCSK2|ESM1	2.4 (1-5.4)	0.038	0.032725399
PCSK2|FGF1	1.8 (1.1-3.1)	0.024	0.022121519
PCSK2|FGF17	1.4 (1-1.9)	0.037	0.035490579
PCSK2|GIPR	1.8 (1-3.1)	0.04	0.036994257
PCSK2|INSL3	2.3 (1.4-4)	0.0024	0.001746285
PCSK2|PROC	2.2 (1.3-3.8)	0.003	0.002284447
PCSK2|PSPN	3 (1.2-7.4)	0.015	0.010896078
PCSK2|TEC	2.6 (1.3-5.4)	0.0077	0.005589722
PCSK2|TNFRSF13C	2.6 (1.2-5.6)	0.013	0.009774717
PDGFD|ESM1	1.7 (1.3-2.4)	0.00078	0.000667398
PDGFD|IL17RA	1.6 (1.1-2.3)	0.0096	0.008945325
PDGFD|JAG2	1.7 (1.1-2.5)	0.016	0.015160476
PDGFD|MAPK8	1.5 (1.1-2.1)	0.02	0.018834377
PDGFD|NR2C1	1.8 (1.3-2.5)	0.00099	0.000845165

PDGFD|TOR2A	1.6 (1.1-2.5)	0.02	0.01833489
PDGFRA|BRD8	1.8 (1.2-2.6)	0.0064	0.005718325
PDGFRA|DHX58	2 (1.4-2.9)	0.00034	0.000256596
PDGFRA|IKBKE	1.9 (1.2-2.9)	0.0029	0.002429421
PDGFRA|NFKBIE	2 (1.1-3.6)	0.032	0.028907962
PDGFRA|PLXNA3	1.5 (1-2.2)	0.031	0.029789779
PDGFRA|PTPN6	2.3 (1.2-4.4)	0.017	0.014495299
PDGFRA|SLC29A3	1.9 (1.2-3)	0.0056	0.004850959
PDGFRA|TAP2	1.8 (1.2-2.7)	0.0041	0.003585929
PDGFRA|TBK1	1.6 (1-2.5)	0.029	0.027909107
PDGFRA|TRIM5	1.7 (1.2-2.4)	0.005	0.00450806
PDGFRA|VAV2	1.9 (1.2-2.9)	0.0058	0.005147444
PIK3R3|RAC3	0.72 (0.51-1)	0.048	0.047477523
PMP2|IL4	1.4 (1-1.9)	0.043	0.042325421
PMP2|PGLYRP2	1.5 (1.1-2.1)	0.0065	0.006126775
PMP2|SLIT1	1.6 (1.2-2.3)	0.0032	0.002909196
PRF1|RAC3	0.57 (0.39-0.84)	0.0041	0.003594378
PRKCB|CCR1	0.49 (0.27-0.91)	0.024	0.020937719
PRKCB|DLL4	0.49 (0.27-0.88)	0.017	0.014660913
PRKCB|FCGR3A	0.11 (0.015-0.76)	0.026	0.006492544
PRKCB|IL27RA	0.3 (0.096-0.94)	0.039	0.028383314
PRKCB|TUBB3	0.71 (0.52-0.99)	0.042	0.040638295
PTH1R|EPOR	2.6 (1.4-4.8)	0.0027	0.00182951
PTH1R|LHB	1.4 (1-1.9)	0.037	0.035834214
PTH1R|LTB4R	2.5 (1.5-4.1)	0.00026	0.000152927
PTH1R|NOD1	2.3 (1.1-4.7)	0.023	0.019669087
RBP4|RAC3	0.44 (0.19-0.99)	0.047	0.040896275
RBP7|BIRC5	1.7 (1-2.8)	0.038	0.035849647
RBP7|PML	1.6 (1.1-2.4)	0.012	0.01145547
ROBO3|ESM1	0.56 (0.35-0.9)	0.016	0.014891639
RORB|CST4	1.5 (1.1-2)	0.018	0.017188468
RORB|GALR2	1.6 (1.1-2.4)	0.015	0.014293226
RORB|KLRD1	1.5 (1.1-2.1)	0.012	0.011943946
RORB|LHB	1.7 (1.1-2.6)	0.021	0.019800473
SEMA3E|CRLF3	2.4 (1.1-5.5)	0.032	0.02704155
SEMA3E|IRF5	3.3 (1.4-7.4)	0.0045	0.002652304
SERPINA3|ESM1	3.4 (1.1-11)	0.038	0.027498691
SFTPD|ESM1	1.5 (1-2.3)	0.037	0.036133187
SLIT2|AGER	1.7 (1.1-2.6)	0.016	0.015054883
SLIT2|APOBEC3F	2 (1.2-3.1)	0.0051	0.00426446
SLIT2|BRD8	9.2 (2.9-29)	0.00017	5.12E-06
SLIT2|CBL	1.8 (1.2-2.9)	0.01	0.009288767
SLIT2|CD72	1.5 (1.1-2.2)	0.014	0.013276235
SLIT2|CRLF3	2 (1-4)	0.039	0.034972961
SLIT2|DHX58	2.5 (1.1-5.6)	0.032	0.02631497
SLIT2|FLT4	1.5 (1.1-2.1)	0.0094	0.008843526
SLIT2|HSPA1L	1.7 (1.2-2.2)	0.0013	0.001124094
SLIT2|IREB2	2.1 (1-4.3)	0.042	0.037737297
SLIT2|IRF5	2.2 (1.1-4.3)	0.024	0.02083233
SLIT2|LTB4R	1.7 (1.2-2.5)	0.0048	0.004282427
SLIT2|MICB	1.6 (1.1-2.5)	0.02	0.018345525
SLIT2|NFATC3	1.9 (1.2-2.9)	0.0077	0.00676191
SLIT2|NMB	3.7 (1.2-12)	0.025	0.016490268
SLIT2|NR2C1	1.8 (1.1-2.9)	0.019	0.017084414
SLIT2|NR2C2	1.9 (1.2-3)	0.011	0.009840048
SLIT2|PDK1	1.5 (1-2.1)	0.028	0.027298906
SLIT2|PIK3CA	1.8 (1.1-3.2)	0.029	0.026453901
SLIT2|PIK3CB	2.3 (1.2-4.6)	0.014	0.010928207

SLIT2|PLXNA3	2.1 (1-4.2)	0.043	0.03855335
SLIT2|RFXAP	2.1 (1.4-3.2)	0.00035	0.000252109
SLIT2|ROBO3	1.4 (1.1-1.9)	0.016	0.015522457
SLIT2|SEMA6C	1.5 (1-2.2)	0.043	0.041479575
SLIT2|SH3BP2	2.2 (1-4.7)	0.042	0.037182611
SLIT2|TRAF3	2.6 (1.3-5.2)	0.0097	0.007333661
TGFB3|PLXNA1	1.5 (1-2.3)	0.03	0.028867662
TGFB3|PLXNA3	1.5 (1.1-2.1)	0.0047	0.004304358
TNC|SPP1	1.6 (1.1-2.3)	0.0095	0.00883235
VIM|CALR	3.6 (1.5-8.9)	0.0049	0.002606837
VIPR2|APOBEC3H	2.3 (1-5.2)	0.048	0.042242512
VIPR2|BMP8A	1.7 (1-2.7)	0.038	0.035548324
VIPR2|CST4	1.6 (1.2-2.2)	0.0028	0.002507667
VIPR2|GIPR	1.9 (1.2-2.9)	0.0047	0.004013068
VIPR2|LTB4R2	1.9 (1.1-3.5)	0.029	0.02637548
VIPR2|NPFF	2.6 (1.3-5.4)	0.0074	0.005368616
VIPR2|NUDT6	1.6 (1-2.6)	0.042	0.040148823
VIPR2|PROC	1.8 (1.1-2.9)	0.015	0.013838925
VIPR2|RLN1	1.5 (1.1-2.2)	0.013	0.012236961
VIPR2|SEMA4G	2.4 (1.2-4.8)	0.017	0.01402212
VIPR2|WFIKKN1	1.5 (1.1-2)	0.022	0.021269291
