# Supplementary material for: Prognostic Implications of Immune-Related Gene Pairs Signatures in Bladder Cancer
Source: J Oncol. 2021 Jul 26;2021:5345181. doi: 10.1155/2021/5345181 (PMC8331311; doi:10.1155/2021/5345181)
Supplement: Supplementary Materials — Supplementary Table 1: 251 IRGPs related to prognosis. Supplementary Table 2: risk score of bladder cancer patients in TCGA dataset and GSE13507 dataset. Supplementary Table 3: mutation frequency of some genes in different risk score groups. Supplementary Table 4: copy number variation of the top 50 genes in different risk score groups. Supplementary Table 5: differentially expressed genes in different risk score groups. [file 5345181.f1.zip › 5345181.f1/Supplementary tables 2 (1).pdf]

Sample	riskScore	risk
TCGA-ZF-A9R7	-1.654794	low
TCGA-K4-A5RH	-1.400165	low
TCGA-K4-A83P	-1.349569	low
TCGA-ZF-AA58	-1.333159	low
TCGA-DK-A3WY	-1.24272	low
TCGA-FD-A62N	-1.241554	low
TCGA-XF-A9SY	-1.240553	low
TCGA-FD-A3NA	-1.235338	low
TCGA-FD-A5BS	-1.216583	low
TCGA-K4-A5RJ	-1.197621	low
TCGA-FD-A43P	-1.175792	low
TCGA-E7-A7PW	-1.106565	low
TCGA-UY-A78O	-1.103959	low
TCGA-UY-A9PB	-1.026442	low
TCGA-CF-A47X	-1.025853	low
TCGA-ZF-A9RF	-1.010525	low
TCGA-ZF-A9R1	-1.008905	low
TCGA-2F-A9KQ	-1.008724	low
TCGA-KQ-A41R	-0.956473	low
TCGA-G2-A2EK	-0.945312	low
TCGA-E5-A4U1	-0.909095	low
TCGA-KQ-A41P	-0.907916	low
TCGA-BT-A42C	-0.907771	low
TCGA-ZF-A9R5	-0.899018	low
TCGA-GD-A3OS	-0.888043	low
TCGA-4Z-AA89	-0.886877	low
TCGA-E7-A8O7	-0.886877	low
TCGA-UY-A78L	-0.882883	low
TCGA-C4-A0F6	-0.878409	low
TCGA-XF-AAMQ	-0.850365	low
TCGA-DK-AA6W	-0.821346	low
TCGA-XF-A9SI	-0.801008	low
TCGA-G2-A2EF	-0.760769	low
TCGA-XF-A9SM	-0.759228	low
TCGA-GV-A3QK	-0.757737	low
TCGA-DK-A3IV	-0.750632	low
TCGA-GC-A3WC	-0.730939	low
TCGA-DK-A3WW	-0.715328	low
TCGA-ZF-AA51	-0.711664	low
TCGA-CF-A3MH	-0.702096	low
TCGA-ZF-A9R4	-0.696487	low
TCGA-ZF-AA4X	-0.68319	low
TCGA-DK-AA6U	-0.679528	low
TCGA-4Z-AA86	-0.67748	low
TCGA-DK-AA74	-0.675024	low
TCGA-ZF-A9RL	-0.667934	low
TCGA-E7-A678	-0.663498	low
TCGA-4Z-AA7N	-0.641762	low
TCGA-GV-A3JX	-0.641114	low
TCGA-FD-A6TB	-0.637727	low
TCGA-G2-AA3D	-0.633495	low
TCGA-E7-A6MF	-0.63153	low
TCGA-UY-A9PD	-0.621493	low
TCGA-DK-A2I4	-0.61718	low
TCGA-XF-A8HB	-0.60699	low
TCGA-GC-A3RB	-0.599151	low
TCGA-LT-A8JT	-0.595964	low

TCGA-GU-A763	-0.595077	low
TCGA-DK-A6B6	-0.586999	low
TCGA-DK-AA76	-0.583895	low
TCGA-UY-A78P	-0.581398	low
TCGA-CU-A3QU	-0.57337	low
TCGA-KQ-A41Q	-0.559861	low
TCGA-FD-A5C1	-0.556694	low
TCGA-SY-A9G5	-0.553516	low
TCGA-XF-A8HC	-0.552301	low
TCGA-H4-A2HQ	-0.55011	low
TCGA-E7-A677	-0.544214	low
TCGA-CF-A47S	-0.541952	low
TCGA-BT-A20P	-0.539263	low
TCGA-KQ-A41O	-0.538458	low
TCGA-DK-AA6X	-0.516139	low
TCGA-E7-A7XN	-0.5084	low
TCGA-GC-A3RD	-0.481951	low
TCGA-ZF-AA4V	-0.480111	low
TCGA-CU-A3KJ	-0.479541	low
TCGA-PQ-A6FI	-0.475762	low
TCGA-GU-AATO	-0.473217	low
TCGA-UY-A9PE	-0.465974	low
TCGA-GV-A40E	-0.462801	low
TCGA-UY-A9PH	-0.460688	low
TCGA-XF-AAN2	-0.444614	low
TCGA-G2-A3IE	-0.439131	low
TCGA-CF-A5UA	-0.438388	low
TCGA-BT-A42F	-0.430879	low
TCGA-FD-A3B6	-0.427333	low
TCGA-K4-A6FZ	-0.425699	low
TCGA-E7-A541	-0.406613	low
TCGA-G2-AA3B	-0.405513	low
TCGA-CF-A9FM	-0.399451	low
TCGA-DK-A6B1	-0.395962	low
TCGA-GD-A6C6	-0.389077	low
TCGA-UY-A9PA	-0.388128	low
TCGA-G2-AA3F	-0.38325	low
TCGA-XF-A9SK	-0.382904	low
TCGA-YF-AA3M	-0.379588	low
TCGA-GC-A3I6	-0.376633	low
TCGA-DK-A1A7	-0.376008	low
TCGA-XF-AAN1	-0.368886	low
TCGA-LT-A5Z6	-0.359522	low
TCGA-DK-A1AA	-0.358327	low
TCGA-BL-A0C8	-0.35641	low
TCGA-XF-AAML	-0.350629	low
TCGA-GD-A2C5	-0.350548	low
TCGA-ZF-A9RM	-0.341563	low
TCGA-GV-A3JZ	-0.337043	low
TCGA-XF-AAN5	-0.337039	low
TCGA-XF-A8HI	-0.321636	low
TCGA-ZF-A9R3	-0.321636	low
TCGA-FJ-A3ZF	-0.319092	low
TCGA-CF-A3MG	-0.316173	low
TCGA-BT-A20J	-0.309592	low
TCGA-ZF-AA53	-0.308553	low
TCGA-CU-A3YL	-0.308245	low
TCGA-DK-A6B0	-0.299939	low

TCGA-CF-A8HX	-0.298845	low
TCGA-DK-AA6T	-0.293855	low
TCGA-XF-AAN0	-0.292733	low
TCGA-E7-A6ME	-0.280102	low
TCGA-FD-A43X	-0.26306	low
TCGA-4Z-AA7Y	-0.253416	low
TCGA-DK-AA6S	-0.251431	low
TCGA-FD-A6TA	-0.249804	low
TCGA-C4-A0F1	-0.245912	low
TCGA-DK-A2I6	-0.244802	low
TCGA-ZF-A9RC	-0.244802	low
TCGA-K4-A54R	-0.244203	low
TCGA-CF-A5U8	-0.243331	low
TCGA-4Z-AA82	-0.24114	low
TCGA-DK-A1AG	-0.239695	low
TCGA-4Z-AA83	-0.233828	low
TCGA-DK-A3IU	-0.23113	low
TCGA-BT-A20Q	-0.219924	low
TCGA-XF-AAMG	-0.217271	low
TCGA-2F-A9KR	-0.213279	low
TCGA-ZF-A9R2	-0.209952	low
TCGA-E5-A2PC	-0.204581	low
TCGA-CU-A0YR	-0.192923	low
TCGA-ZF-A9RN	-0.183127	low
TCGA-G2-A3VY	-0.180391	low
TCGA-DK-A6B5	-0.175516	low
TCGA-CF-A3MI	-0.169376	low
TCGA-UY-A8OB	-0.168936	low
TCGA-4Z-AA7W	-0.165754	low
TCGA-BT-A3PJ	-0.165385	low
TCGA-ZF-A9RD	-0.158276	low
TCGA-FD-A6TE	-0.155865	low
TCGA-4Z-AA7O	-0.155207	low
TCGA-DK-A1A5	-0.149886	low
TCGA-CF-A8HY	-0.142201	low
TCGA-BT-A20W	-0.138702	low
TCGA-DK-A1AE	-0.13751	low
TCGA-XF-AAN3	-0.133471	low
TCGA-CU-A5W6	-0.132284	high
TCGA-XF-A9T5	-0.13124	high
TCGA-YF-AA3L	-0.121936	high
TCGA-XF-A9SH	-0.120443	high
TCGA-4Z-AA7M	-0.107974	high
TCGA-H4-A2HO	-0.106721	high
TCGA-E7-A519	-0.106555	high
TCGA-FJ-A871	-0.106106	high
TCGA-4Z-AA7S	-0.105972	high
TCGA-GD-A3OQ	-0.102806	high
TCGA-CF-A47V	-0.097437	high
TCGA-XF-A9SX	-0.096688	high
TCGA-XF-A8HF	-0.096663	high
TCGA-BT-A20N	-0.096372	high
TCGA-BT-A20O	-0.086454	high
TCGA-DK-AA6L	-0.08404	high
TCGA-4Z-AA81	-0.074161	high
TCGA-GU-A42R	-0.072589	high
TCGA-GU-A766	-0.071949	high
TCGA-XF-A8HD	-0.066024	high

TCGA-DK-A2I1	-0.063751	high
TCGA-GV-A3QH	-0.061506	high
TCGA-GV-A40G	-0.061428	high
TCGA-DK-A1AC	-0.058883	high
TCGA-DK-A3IK	-0.053456	high
TCGA-UY-A8OD	-0.053176	high
TCGA-DK-AA77	-0.048715	high
TCGA-G2-A2ES	-0.048419	high
TCGA-BT-A42E	-0.047542	high
TCGA-DK-AA6P	-0.043928	high
TCGA-GU-A764	-0.043542	high
TCGA-GV-A6ZA	-0.042219	high
TCGA-HQ-A2OE	-0.034718	high
TCGA-LC-A66R	-0.027253	high
TCGA-GC-A3BM	-0.026724	high
TCGA-CF-A27C	-0.015664	high
TCGA-MV-A51V	-0.011733	high
TCGA-ZF-AA4T	-0.010095	high
TCGA-S5-AA26	-0.009729	high
TCGA-E7-A3X6	-0.00754	high
TCGA-E7-A4XJ	-0.003396	high
TCGA-CF-A47T	-0.002659	high
TCGA-CF-A3MF	0	high
TCGA-YC-A89H	0	high
TCGA-GU-AATP	0	high
TCGA-2F-A9KT	0	high
TCGA-DK-A3X1	0	high
TCGA-XF-A9T0	0	high
TCGA-FD-A3SN	0.0036621	high
TCGA-FD-A3SP	0.0170648	high
TCGA-GU-A42P	0.0257531	high
TCGA-4Z-AA7Q	0.0319367	high
TCGA-FD-A43U	0.0320567	high
TCGA-CF-A47W	0.0377212	high
TCGA-UY-A78M	0.0420758	high
TCGA-GC-A3YS	0.0420758	high
TCGA-FD-A62O	0.0504145	high
TCGA-UY-A9PF	0.0571547	high
TCGA-K4-A3WU	0.0590628	high
TCGA-FD-A3N6	0.0630947	high
TCGA-GV-A3JW	0.0641586	high
TCGA-FD-A5BY	0.0652089	high
TCGA-G2-A2EO	0.0692807	high
TCGA-E7-A97P	0.0726011	high
TCGA-FD-A6TC	0.0766724	high
TCGA-FT-A3EE	0.0771074	high
TCGA-4Z-AA7R	0.0827813	high
TCGA-XF-A9T8	0.0849208	high
TCGA-5N-A9KI	0.0851784	high
TCGA-CU-A0YO	0.0938136	high
TCGA-XF-AAMR	0.0942709	high
TCGA-GU-A762	0.0946135	high
TCGA-YC-A8S6	0.0970102	high
TCGA-FD-A5BT	0.1038078	high
TCGA-4Z-AA87	0.1041438	high
TCGA-DK-A6B2	0.1045887	high
TCGA-CF-A9FF	0.1072706	high
TCGA-DK-A6AV	0.1093466	high

TCGA-ZF-AA5P	0.1110911	high
TCGA-FD-A6TD	0.1210329	high
TCGA-BT-A3PH	0.1227838	high
TCGA-DK-A3IS	0.1293056	high
TCGA-E7-A85H	0.1305632	high
TCGA-E7-A4IJ	0.1361461	high
TCGA-K4-AAQO	0.1435017	high
TCGA-PQ-A6FN	0.1462196	high
TCGA-CF-A1HR	0.1488173	high
TCGA-DK-A6AW	0.1558804	high
TCGA-DK-A3X2	0.1585446	high
TCGA-E7-A6MD	0.1646615	high
TCGA-DK-AA71	0.1692851	high
TCGA-FD-A3N5	0.171364	high
TCGA-DK-A1AD	0.1722008	high
TCGA-GV-A3QI	0.1742223	high
TCGA-4Z-AA84	0.1816182	high
TCGA-FD-A5C0	0.1851586	high
TCGA-GC-A3RC	0.195774	high
TCGA-BT-A0YX	0.1959733	high
TCGA-FJ-A3Z9	0.1981182	high
TCGA-XF-A9SZ	0.2021447	high
TCGA-BT-A2LD	0.2030554	high
TCGA-E7-A7DV	0.2069845	high
TCGA-K4-A5RI	0.2071754	high
TCGA-DK-A1A6	0.2077617	high
TCGA-XF-A9T4	0.2126821	high
TCGA-CF-A7I0	0.2146234	high
TCGA-DK-AA6Q	0.2160569	high
TCGA-ZF-AA4R	0.2320388	high
TCGA-XF-A9SU	0.2325401	high
TCGA-CF-A9FL	0.2343038	high
TCGA-BT-A3PK	0.2376711	high
TCGA-5N-A9KM	0.2378656	high
TCGA-FJ-A3Z7	0.2404176	high
TCGA-2F-A9KO	0.2543106	high
TCGA-XF-A9SJ	0.2587203	high
TCGA-K4-A3WS	0.2619527	high
TCGA-DK-AA6R	0.2641572	high
TCGA-G2-A2EC	0.2718802	high
TCGA-DK-AA6M	0.2729929	high
TCGA-FD-A3B8	0.2735161	high
TCGA-R3-A69X	0.2755363	high
TCGA-FD-A6TK	0.2931979	high
TCGA-XF-AAME	0.2970903	high
TCGA-BT-A20T	0.2973823	high
TCGA-FD-A5BZ	0.2991543	high
TCGA-G2-A2EJ	0.3077195	high
TCGA-FD-A5BV	0.3082105	high
TCGA-XF-AAMZ	0.3103661	high
TCGA-FD-A3B3	0.3129119	high
TCGA-ZF-AA54	0.3155427	high
TCGA-BL-A13I	0.3188488	high
TCGA-K4-A3WV	0.3231763	high
TCGA-XF-AAMT	0.3252566	high
TCGA-ZF-AA4U	0.3424019	high
TCGA-XF-A8HE	0.344804	high
TCGA-FD-A43Y	0.3448128	high

TCGA-ZF-AA5H	0.346098	high
TCGA-GD-A3OP	0.3479537	high
TCGA-FD-A62S	0.3491976	high
TCGA-UY-A78K	0.3610038	high
TCGA-BT-A20V	0.3616917	high
TCGA-2F-A9KP	0.3629851	high
TCGA-DK-A3IT	0.3640413	high
TCGA-HQ-A5NE	0.364704	high
TCGA-FD-A43S	0.3759338	high
TCGA-SY-A9G0	0.3869118	high
TCGA-ZF-A9R0	0.3994866	high
TCGA-XF-A8HG	0.4040732	high
TCGA-FD-A5BU	0.4152744	high
TCGA-FD-A6TG	0.4331211	high
TCGA-BT-A20U	0.4356903	high
TCGA-ZF-AA5N	0.4368699	high
TCGA-ZF-A9R9	0.4393908	high
TCGA-BT-A2LB	0.4406596	high
TCGA-2F-A9KW	0.4423201	high
TCGA-KQ-A41S	0.4429656	high
TCGA-FD-A43N	0.4491607	high
TCGA-E7-A3Y1	0.4509877	high
TCGA-FD-A3B7	0.4862371	high
TCGA-E7-A97Q	0.4886864	high
TCGA-GV-A3QF	0.4946613	high
TCGA-FD-A6TH	0.5182699	high
TCGA-G2-AA3C	0.5193178	high
TCGA-FT-A61P	0.5199387	high
TCGA-FD-A3SQ	0.5201879	high
TCGA-FD-A5BR	0.5243142	high
TCGA-DK-A1A3	0.5245283	high
TCGA-XF-A8HH	0.5355425	high
TCGA-ZF-A9RE	0.5364388	high
TCGA-FD-A3SJ	0.54887	high
TCGA-CF-A47Y	0.5592821	high
TCGA-ZF-AA56	0.5599319	high
TCGA-UY-A78N	0.56431	high
TCGA-GV-A3JV	0.56935	high
TCGA-CU-A72E	0.5706651	high
TCGA-DK-A2HX	0.5801674	high
TCGA-CU-A0YN	0.5860145	high
TCGA-E5-A4TZ	0.5972899	high
TCGA-XF-AAMW	0.5973035	high
TCGA-XF-AAMH	0.5988593	high
TCGA-DK-A3IN	0.6111776	high
TCGA-DK-A3IM	0.6180774	high
TCGA-DK-A1AF	0.6229763	high
TCGA-KQ-A41N	0.6368157	high
TCGA-K4-A6MB	0.638524	high
TCGA-GC-A3OO	0.6399926	high
TCGA-G2-A3IB	0.6403924	high
TCGA-DK-A3WX	0.6404767	high
TCGA-XF-AAMX	0.646483	high
TCGA-ZF-AA52	0.6464889	high
TCGA-G2-A2EL	0.6496605	high
TCGA-XF-A9SV	0.6598638	high
TCGA-ZF-AA4W	0.6629172	high
TCGA-XF-AAMY	0.6697096	high

TCGA-DK-A3IL	0.6958095	high
TCGA-XF-A9SP	0.7024101	high
TCGA-K4-A4AC	0.7206772	high
TCGA-HQ-A5ND	0.7277925	high
TCGA-FD-A3SO	0.7754446	high
TCGA-FJ-A3ZE	0.7789364	high
TCGA-FD-A3SR	0.7836594	high
TCGA-XF-AAN4	0.7888632	high
TCGA-BL-A13J	0.7930599	high
TCGA-FD-A3SM	0.7945215	high
TCGA-C4-A0F0	0.798458	high
TCGA-BT-A20R	0.8088536	high
TCGA-DK-AA75	0.8096182	high
TCGA-XF-A9ST	0.8101741	high
TCGA-FD-A3B4	0.820586	high
TCGA-FD-A3B5	0.8260337	high
TCGA-XF-A9T6	0.8634009	high
TCGA-XF-AAN7	0.8729027	high
TCGA-FD-A3SL	0.8965328	high
TCGA-GU-AATQ	0.9110535	high
TCGA-BL-A3JM	0.9151784	high
TCGA-S5-A6DX	0.9252911	high
TCGA-BT-A2LA	0.9343419	high
TCGA-XF-A9T2	0.9394688	high
TCGA-BL-A5ZZ	0.9998257	high
TCGA-DK-A1AB	1.0077942	high
TCGA-BT-A20X	1.0170706	high
TCGA-FD-A5BX	1.028943	high
TCGA-DK-A2I2	1.0375532	high
TCGA-XF-AAMJ	1.0611915	high
TCGA-GU-A767	1.1485111	high
TCGA-XF-AAN8	1.1854173	high
TCGA-FD-A3SS	1.2267445	high
TCGA-FD-A6TF	1.2742022	high
TCGA-GU-A42Q	1.2864745	high
TCGA-DK-A3IQ	1.3196576	high
TCGA-FD-A62P	1.364213	high
TCGA-FD-A6TI	1.4374702	high
TCGA-XF-A9T3	1.4511335	high
TCGA-C4-A0EZ	1.4529415	high
TCGA-XF-A9SW	1.6320684	high
TCGA-XF-A9SL	1.7809643	high
TCGA-C4-A0F7	1.86967	high
TCGA-BT-A0S7	1.9658169	high
