# Supplementary material for: Prognostic Implications of Immune-Related Gene Pairs Signatures in Bladder Cancer
Source: J Oncol. 2021 Jul 26;2021:5345181. doi: 10.1155/2021/5345181 (PMC8331311; doi:10.1155/2021/5345181)
Supplement: Supplementary Materials — Supplementary Table 1: 251 IRGPs related to prognosis. Supplementary Table 2: risk score of bladder cancer patients in TCGA dataset and GSE13507 dataset. Supplementary Table 3: mutation frequency of some genes in different risk score groups. Supplementary Table 4: copy number variation of the top 50 genes in different risk score groups. Supplementary Table 5: differentially expressed genes in different risk score groups. [file 5345181.f1.zip › 5345181.f1/Supplementary tables 3 (1).pdf]

Gene	low risksco	high risksc	Mutation difference
EP300	18%	11%	-7%
ERBB2	14%	8%	-6%
SYNE1	21%	15%	-6%
LRP1B	13%	7%	-6%
KDM6A	28%	23%	-5%
PIK3CA	23%	18%	-5%
FLG	16%	11%	-5%
AHNAK	12%	7%	-5%
TTN	44%	39%	-5%
CSMD3	13%	9%	-4%
MUC16	27%	23%	-4%
ADGRV1	12%	8%	-4%
CDKN1A	12%	8%	-4%
KMT2A	12%	8%	-4%
AKAP9	11%	8%	-3%
BIRC6	11%	8%	-3%
ELF3	13%	10%	-3%
ERBB3	11%	8%	-3%
RYR2	16%	13%	-3%
SYNE2	11%	8%	-3%
STAG2	14%	12%	-2%
CUBN	12%	10%	-2%
FGFR3	15%	13%	-2%
RB1	19%	18%	-1%
NEB	11%	10%	-1%
AHNAK2	10%	10%	0%
CREBBP	10%	10%	0%
OBSCN	10%	11%	1%
SPTAN1	10%	11%	1%
ATM	12%	13%	1%
FAT4	12%	13%	1%
TP53	47%	48%	1%
HMCN1	14%	16%	2%
KMT2D	25%	27%	2%
KMT2C	12%	16%	4%
ARID1A	20%	27%	7%
MACF1	9%	18%	9%
