# Supplementary material for: Prognostic Implications of Immune-Related Gene Pairs Signatures in Bladder Cancer
Source: J Oncol. 2021 Jul 26;2021:5345181. doi: 10.1155/2021/5345181 (PMC8331311; doi:10.1155/2021/5345181)
Supplement: Supplementary Materials — Supplementary Table 1: 251 IRGPs related to prognosis. Supplementary Table 2: risk score of bladder cancer patients in TCGA dataset and GSE13507 dataset. Supplementary Table 3: mutation frequency of some genes in different risk score groups. Supplementary Table 4: copy number variation of the top 50 genes in different risk score groups. Supplementary Table 5: differentially expressed genes in different risk score groups. [file 5345181.f1.zip › 5345181.f1/Supplementary tables 4 (1).pdf]

Gene	LOSS	LOSS_freq	GAIN	GAIN_freq	CNV	CNV_freq	Wild	Wild_freq
MED30	0	0	37	0.24183	37	0.24183	116	0.75817
EXOC6B	2	0.013072	6	0.039216	8	0.052288	145	0.947712
COPS5	0	0	25	0.163399	25	0.163399	128	0.836601
SGK3	0	0	25	0.163399	25	0.163399	128	0.836601
USP53	12	0.078431	6	0.039216	18	0.117647	135	0.882353
STMN1	6	0.039216	8	0.052288	14	0.091503	139	0.908497
MCMDC2	0	0	25	0.163399	25	0.163399	128	0.836601
GGA2	11	0.071895	12	0.078431	23	0.150327	130	0.849673
EARS2	11	0.071895	12	0.078431	23	0.150327	130	0.849673
ZNF436	5	0.03268	11	0.071895	16	0.104575	137	0.895425
TCF24	0	0	25	0.163399	25	0.163399	128	0.836601
HNRNPR	5	0.03268	11	0.071895	16	0.104575	137	0.895425
SPR	2	0.013072	5	0.03268	7	0.045752	146	0.954248
ERN2	12	0.078431	11	0.071895	23	0.150327	130	0.849673
PRKCB	15	0.098039	12	0.078431	27	0.176471	126	0.823529
UBFD1	11	0.071895	12	0.078431	23	0.150327	130	0.849673
PALB2	12	0.078431	12	0.078431	24	0.156863	129	0.843137
NDUFAB1	11	0.071895	12	0.078431	23	0.150327	130	0.849673
FABP2	11	0.071895	6	0.039216	17	0.111111	136	0.888889
EMX1	2	0.013072	5	0.03268	7	0.045752	146	0.954248
DCTN5	12	0.078431	10	0.065359	22	0.143791	131	0.856209
AARD	0	0	38	0.248366	38	0.248366	115	0.751634
AIFM1	2	0.013072	0	0	2	0.013072	151	0.986928
RAD21	0	0	37	0.24183	37	0.24183	116	0.75817
TRPS1	0	0	37	0.24183	37	0.24183	116	0.75817
PDE5A	11	0.071895	6	0.039216	17	0.111111	136	0.888889
MAD2L1	11	0.071895	6	0.039216	17	0.111111	136	0.888889
PPP1R42	0	0	25	0.163399	25	0.163399	128	0.836601
C4orf3	11	0.071895	6	0.039216	17	0.111111	136	0.888889
CNOT6L	7	0.045752	10	0.065359	17	0.111111	136	0.888889
LUZP1	6	0.039216	8	0.052288	14	0.091503	139	0.908497
CHP2	12	0.078431	11	0.071895	23	0.150327	130	0.849673
LIMCH1	16	0.104575	6	0.039216	22	0.143791	131	0.856209
PRDM5	13	0.084967	6	0.039216	19	0.124183	134	0.875817
CACNG3	12	0.078431	13	0.084967	25	0.163399	128	0.836601
TCEA3	5	0.03268	10	0.065359	15	0.098039	138	0.901961
AUNIP	6	0.039216	8	0.052288	14	0.091503	139	0.908497
TRAPPC9	2	0.013072	33	0.215686	35	0.228758	118	0.771242
SLC30A8	0	0	38	0.248366	38	0.248366	115	0.751634
EIF3H	1	0.006536	37	0.24183	38	0.248366	115	0.751634
UTP23	0	0	37	0.24183	37	0.24183	116	0.75817
MYOZ2	11	0.071895	6	0.039216	17	0.111111	136	0.888889
ZDHHC9	2	0.013072	0	0	2	0.013072	151	0.986928
COG7	11	0.071895	12	0.078431	23	0.150327	130	0.849673
EDA2R	0	0	1	0.006536	1	0.006536	152	0.993464
PAQR7	6	0.039216	8	0.052288	14	0.091503	139	0.908497
PLK1	12	0.078431	10	0.065359	22	0.143791	131	0.856209
AC087350	0	0	37	0.24183	37	0.24183	116	0.75817
C8orf44-S	0	0	25	0.163399	25	0.163399	128	0.836601
