# Supplementary material for: Prognostic Implications of Immune-Related Gene Pairs Signatures in Bladder Cancer
Source: J Oncol. 2021 Jul 26;2021:5345181. doi: 10.1155/2021/5345181 (PMC8331311; doi:10.1155/2021/5345181)
Supplement: Supplementary Materials — Supplementary Table 1: 251 IRGPs related to prognosis. Supplementary Table 2: risk score of bladder cancer patients in TCGA dataset and GSE13507 dataset. Supplementary Table 3: mutation frequency of some genes in different risk score groups. Supplementary Table 4: copy number variation of the top 50 genes in different risk score groups. Supplementary Table 5: differentially expressed genes in different risk score groups. [file 5345181.f1.zip › 5345181.f1/Supplementary tables 5 (1).pdf]

Gene	logFC	AveExpr	t	P.Value	adj.P.Val	B
CRYAB	1.144465	3.458451	7.128953	4.94E-12	2.10E-08	16.86721
FLNC	1.326977	2.693244	7.093427	6.20E-12	2.10E-08	16.64952
HSPB6	1.437547	3.192875	6.949472	1.55E-11	3.27E-08	15.77597
ACTA2	1.216509	7.112371	6.807069	3.77E-11	5.98E-08	14.9255
COL1A1	1.36713	8.99134	6.555076	1.77E-10	1.36E-07	13.45444
TAGLN	1.255464	6.823945	6.532055	2.03E-10	1.47E-07	13.32224
COL6A3	1.147912	5.380674	6.504965	2.39E-10	1.56E-07	13.16716
ELN	1.109876	3.339297	6.48817	2.64E-10	1.60E-07	13.07127
COL5A1	1.185553	5.428316	6.487174	2.66E-10	1.60E-07	13.06558
CCN2	1.153324	6.506918	6.440108	3.52E-10	2.03E-07	12.79795
CNN1	1.568201	4.426589	6.408512	4.24E-10	2.34E-07	12.61916
BGN	1.034537	8.137352	6.394556	4.61E-10	2.47E-07	12.54042
PLN	1.185762	2.289658	6.375695	5.15E-10	2.61E-07	12.43421
ACTG2	1.584214	5.229125	6.317687	7.25E-10	3.60E-07	12.10917
MYL9	1.100879	7.231437	6.303027	7.90E-10	3.85E-07	12.0274
COL3A1	1.314617	8.695405	6.249676	1.08E-09	4.88E-07	11.73114
EMILIN1	1.081906	5.089496	6.155113	1.87E-09	6.75E-07	11.21105
FHL1	1.073954	3.173869	6.115165	2.35E-09	8.25E-07	10.99329
COL1A2	1.22043	8.079784	6.086844	2.76E-09	9.04E-07	10.83961
CCDC80	1.07135	3.212447	6.085228	2.78E-09	9.04E-07	10.83086
AEBP1	1.250274	6.299098	6.07412	2.96E-09	9.27E-07	10.77076
COL5A2	1.027031	5.562624	6.066862	3.09E-09	9.38E-07	10.73154
SMOC2	1.020957	3.429371	6.06557	3.11E-09	9.38E-07	10.72457
FN1	1.3595	7.08883	6.034321	3.72E-09	1.09E-06	10.55617
LMOD1	1.161049	3.51998	5.967001	5.43E-09	1.46E-06	10.19583
COL6A1	1.015297	7.175668	5.941758	6.26E-09	1.62E-06	10.06158
COL6A2	1.078144	7.458816	5.933509	6.55E-09	1.68E-06	10.01781
SFRP2	1.910747	5.092033	5.911561	7.41E-09	1.82E-06	9.901596
DCN	1.119864	4.855471	5.861963	9.75E-09	2.21E-06	9.640302
POSTN	1.466399	5.331426	5.845826	1.07E-08	2.35E-06	9.555684
DES	2.0895	5.11604	5.777466	1.55E-08	3.20E-06	9.199385
ASPN	1.058963	3.36774	5.775972	1.57E-08	3.20E-06	9.191636
PTGIS	1.153039	2.761647	5.650588	3.09E-08	5.00E-06	8.547417
CTHRC1	1.112197	5.393619	5.505406	6.69E-08	9.26E-06	7.816421
MYH11	1.375275	3.93635	5.500347	6.87E-08	9.46E-06	7.791238
CHRDL2	1.097187	2.192053	5.460375	8.48E-08	1.11E-05	7.59298
CPXM1	1.012175	4.091051	5.432811	9.79E-08	1.25E-05	7.456981
LUM	1.050665	7.276024	5.41161	1.09E-07	1.37E-05	7.352778
MFAP4	1.089676	5.186935	5.362217	1.41E-07	1.66E-05	7.111366
IGFBP5	1.083425	5.796255	5.277892	2.17E-07	2.31E-05	6.703619
ISLR	1.177087	5.001571	5.264332	2.33E-07	2.46E-05	6.63857
SFRP4	1.450406	3.519807	5.25351	2.46E-07	2.57E-05	6.586763
EFEMP1	1.149031	3.905715	5.191325	3.37E-07	3.24E-05	6.290841
HTRA3	1.042469	4.675211	5.122567	4.75E-07	4.22E-05	5.9672
ACTC1	1.171944	1.890649	5.119017	4.83E-07	4.28E-05	5.950595
COMP	1.336207	3.719998	5.065915	6.29E-07	5.26E-05	5.703367
TNC	1.026877	4.197744	4.906879	1.36E-06	9.30E-05	4.976463
CTSE	-1.26265	2.9128	-4.65039	4.54E-06	0.000242	3.847328
CLIC3	1.046766	5.507042	4.444008	1.15E-05	0.000516	2.978089
PCP4	1.081244	2.272345	4.32824	1.91E-05	0.000752	2.506066
PTN	1.21381	4.726813	4.323989	1.95E-05	0.000762	2.488948
TRIM31	-1.06199	3.033632	-4.29352	2.22E-05	0.00084	2.366704
CCL21	1.102157	3.379016	4.240858	2.78E-05	0.001002	2.157261
CES1	1.019944	2.663924	4.21311	3.13E-05	0.001099	2.047853
SPRR1B	1.15807	4.513305	3.391517	0.000766	0.013247	-0.89025
